# Bader’s Topological Bond Path Does Not Necessarily Indicate Stabilizing Interaction—Proof Studies Based on the Ng@[3_n_]cyclophane Endohedral Complexes

**DOI:** 10.3390/molecules28176353

**Published:** 2023-08-30

**Authors:** Mirosław Jabłoński

**Affiliations:** Faculty of Chemistry, Nicolaus Copernicus University, Gagarina 7, 87-100 Toruń, Poland; teojab@chem.umk.pl; Tel.: +48-056-611-4695

**Keywords:** superphane, cyclophane, noble gas atom, endohedral complex, exohedral complex, encapsulation, steric crowding, repulsion, QTAIM, bond path

## Abstract

According to Bader’s quantum theory of atoms in molecules (QTAIM), the simultaneous presence of a bond path and the corresponding bond critical point between any two atoms is both a necessary and sufficient condition for the atoms to be bonded to one another. In principle, this means that this pair of atoms should make a stabilizing contribution to the molecular system. However, the multitude of so-called counterintuitive bond paths strongly suggests that this statement is not necessarily true. Particularly ‘troublesome’ are endohedral complexes, in which encapsulation-enforced proximity between the trapped guest (e.g., an atom) and the host’s cage system usually ‘produces’ many counterintuitive bond paths. In the author’s opinion, the best evidence to demonstrate the repulsive nature of the intra-cage guest⋯host interaction is the use of some trapping systems containing small escape channels and then showing that the initially trapped entity spontaneously escapes outside the host’s cage during geometry optimization of the initially built guest@host endohedral complex. For this purpose, a group of 24 Ng@[3${}_{n}$]cyclophane (3≤n≤6) endohedral complexes is used. As a result, arguments are presented showing that Bader’s topological bond path does not necessarily indicate a stabilizing interaction.

## 1. Introduction

Chemists are eager to see atoms and bonds in molecules, which is why they enthusiastically embraced the quantum theory of atoms in molecules (QTAIM) [1,2,3] created by Richard F.W. Bader. This theory, in a simple and visual way, enables the division of a molecule into individual atoms and introduces the concept of the so-called bond path (BP). Bond paths together with the corresponding bond critical points (BCPs) form a molecular graph, which is a characteristic topological imprint of a given molecule resulting from the appropriate distribution of its electron density in real three-dimensional space. According to QTAIM, the simultaneous presence of BP and the corresponding BCP between any two atoms is both a necessary and sufficient condition for the atoms to be bonded to one another when there are no net forces acting on the nuclei [1]. Importantly, it seems that in the early years of QTAIM, Bader treated bond paths as identifiers for chemical bonds [4,5,6,7]. Moreover, it turned out that in a huge number of molecules (albeit rather simple ones) the molecular graphs were consistent with the structural formulas of these molecules [8]. This finding greatly encouraged the chemist community to use QTAIM, or more specifically, the presence of a bond path, as a tool to confirm (or disprove) the presence of various chemical bonds. Moreover, the same approach is often used to look for much weaker interatomic interactions such as, e.g., intermolecular hydrogen bonds, and is often taken as evidence of their presence.

Unfortunately, the situation quickly began to become complicated and became more and more unclear. In 1991, Cioslowski et al. [9] obtained as many as 30 BPs between the Ne atom trapped in the C${}_{60}$ fullerene and the C=C bonds. For this reason, they suggested that these BPs result from steric interactions. Shortly afterwards, theoretically examining the interaction between ortho-substituted hydrogens in biphenyl, Cioslowski and Mixon suggested that BPs delineate only major, but not necessarily bonding, interactions present within a given chemical system [10]. Moreover, they suggested for the first time that BCPs do not necessarily indicate attractive (bonding) interactions, as BPs describing nonbonding steric interactions are common in molecules with close atomic proximity [10]. As such, BPs are specifically expected to be generated in sterically crowded systems; endohedral complexes have proven to be particularly important and interesting chemical systems for further investigation of this issue.

It took more than ten years for the topic of ‘repulsive bond paths’ to be taken up by others as well. Both importantly and interestingly, Cerpa et al. [11] obtained 8 and 20 BPs between the He atom and carbon atoms in the He@cubane and He@C${}_{20}$H${}_{20}$ endohedral complexes, respectively, while the number of Ng⋯C (Ng = noble gas atom) bond paths in Ng@C${}_{60}$ was as high as 60. They therefore showed that the large amount of BPs may simply be due to the high symmetry of the system. Consequently, they concluded that “the number of gradient paths terminating at an atom is chemically meaningless” and, therefore, “it is risky to make the one-to-one analogy between a bond path and a chemical bond in the usual chemical sense of the word” [11]. Around the same time, Haaland et al. [12,13] demonstrated the antibonding nature of the He⋯C interactions (traced by the He⋯C BPs) in the He@adamantane complex by obtaining the negative dissociation energy of this complex. They concluded that “the spontaneous dissociation of He@adamantane is prevented solely by the rigidity of the adamantane framework” [12,13]. This fact has been shown even better by Poater et al. [14] by a modeled removal of one of the –CH${}_{2}$– bridges, which resulted in the spontaneous escape of the He atom from the adamantane interior. Consequently, they concluded that the He atom is trapped in the adamantane cage structure not because the He⋯C interaction is binding, but quite the contrary, because the He⋯C repulsion prevents the He atom from escaping. For this reason, the interpretation of the presence of BPs and BCPs as indicators of bonding is incorrect [14]. By obtaining a negative value of the dissociation energy (similarly to Haaland et al. for He@adamantane [12,13]) of the reaction He${}_{2}$@C${}_{20}$H${}_{20}$ → He${}_{2}$ + C${}_{20}$H${}_{20}$ Cerpa et al. [15] demonstrated the repulsive nature of the He${}_{2}\cdots$C${}_{20}$H${}_{20}$ interactions in the endohedral He${}_{2}$@C${}_{20}$H${}_{20}$ complex. Moreover, this result was confirmed by obtaining a large positive value of the interaction energy, which resulted from the significant predominance of the Pauli energy over the electrostatic component. A little earlier, in 2007, in the same way, Krapp and Frenking showed the repulsive nature of the Ng${}_{2}\cdots$C${}_{60}$ interactions in the Ng${}_{2}$@C${}_{60}$ complex. In the case of larger Ng atoms (Ar, Kr, Xe), they obtained six BPs of the type Ng⋯C [16]. It is also worth mentioning the article by Moran et al. from 2003 [17], in which they studied the energetics of various endohedral and exohedral complexes, coming to the conclusion that “exohedral binding is preferred to endohedral encapsulation without exception”.

It should be noted that the presence of unexpected (counterintuitive) bond paths has been found in many molecular systems. Although they occur most often between two closely electronegative atoms [9,18,19,20,21,22,23,24,25,26,27,28,29,30,31,32,33,34,35,36,37], especially those with large radii [21,33,34], another important group are H⋯H bond paths [10,14,38,39,40,41,42,43]. For numerous examples of systems with counterintuitive bond paths, see references [33,34,35]. Therefore, the described endohedral complexes are just another group of systems featuring counterintuitive bond paths.

In response to the increasing number of examples of the presence of counterintuitive bond paths, as well as attacks on the interpretation of a bond path as indicative of a chemical bond or necessarily a stabilizing interaction, Bader has launched a defensive article in which he has emphasized that bond paths should not be considered as chemical bonds [44]. Anyway, the author of this article is of the opinion that a pair of atoms forming a bond or being ‘bonded to one another’ (to use the newer phrase of Bader [1]) should contribute to the stabilization of the molecule. This is consistent with the earlier statement by Strenalyuk and Haaland that “it is the attractive interactions that lead to the formation of an aggregation, while the repulsive interactions oppose it” [13].

**Figure 1:**
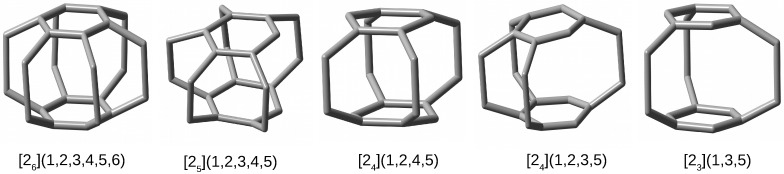
Structures (hydrogen atoms have been removed for better visualization of the carbon backbones) of the most important [2${}_{n}$]cyclophanes investigated in reference [52].

The previously studied cagey systems (e.g., tetrahedrane, cubane, adamantane, dodecahedrane, buckminsterfullerene) have too rigid carbon skeletons to clearly show the influence of a trapped atom on their structure. Moreover, this rigidity together with their closed structure prevents the initially trapped atom from spontaneously escaping outside the cage of the trapping molecule (i.e., the host). Nevertheless, I have recently used [2${}_{6}$](1,2,3,4,5,6)cyclophane, i.e., [2${}_{6}$]SP (SP = superphane) [45,46,47,48,49] (see Figure 1), belonging to a wide group of cyclophanes [50], showing that its carbon skeleton is flexible enough in order to clearly demonstrate the effect of the Ng atom trapped in [2${}_{6}$]SP on the structure of a cagey molecule [51]. Namely, it has been shown that the encapsulation of Ng in the interior of the [2${}_{6}$]SP molecule leads to a strong “swelling” of [2${}_{6}$]SP, visible as a significant increase in the intercarbon distances, especially the distance between two benzene rings. Despite this swelling, the fairly compact structure of the [2${}_{6}$]SP molecule resulting from the presence of as many as six ethylene bridges does not allow the trapped Ng atom (but Kr on some levels of theory) to escape from the [2${}_{6}$]SP cage [51]. Therefore, I have then studied [52] a series of endohedral complexes involving various cyclophanes derived from [2${}_{6}$]SP, in which the number of the side ethylene bridges was reduced to 2 ≤*n*≤ 5 (i.e., [2${}_{n}$]CP [53,54,55,56,57,58,59,60,61,62,63,64,65]). It has turned out that the presence of at least one one-carbon window in the side skeleton of cyclophane (as is the case with cyclophanes [2${}_{5}$](1,2,3,4,5), [2${}_{4}$](1,2,4,5), [2${}_{4}$](1,2,3,5), [2${}_{3}$](1,3,5); see Figure 1) in most cases leads to the escape of the initially trapped Ng atom outside the cage, creating a much more energetically stable cyclophane⋯Ng exohedral complex. Only in the case of the smallest He atom did this atom remain inside the considered [2${}_{n}$]cyclophanes, forming He@[2${}_{n}$]CP endohedral complexes [52]. However, as shown, these complexes were always much less energetically favorable than the exohedral complexes [2${}_{n}$]CP⋯He. Thus, this result has shown the repulsive nature of the Ng⋯C interactions in the endohedral complexes. Due to the presence of Ng⋯C bond paths, this result is also strong proof that the presence of a bond path is not an indicator that a given interaction is attractive [52].

In this article, I have described the effect of the Ng atom encapsulation in slightly larger [3${}_{n}$]cyclophanes [66,67,68,69,70,71,72,73,74,75,76,77] on their structure, and above all on energetical aspects. It is essential to investigate the character of the Ng⋯C interaction between the trapped Ng atom and the interior of the trapping molecule. Demonstration of its destabilizing (repulsive) nature together with the possible presence of the Ng⋯C bond path will be another example proving that a bond path does not necessarily indicate, as many still believe, the binding nature of a given interaction.

The presence of longer site bridges, trimethylene instead of ethylene, creates new intriguing possibilities for the Ng atom initially trapped inside [3${}_{n}$]CP. Namely, on the one hand, the larger internal space of [3${}_{n}$]CP compared to [2${}_{n}$]CP should lower the internal Ng⋯C repulsion, and thus facilitate the Ng atom inside the [3${}_{n}$]CP molecule to form an endohedral complex Ng@[3${}_{n}$]CP; however, on the other hand, the larger one-carbon window (which can be seen as an exit/escape channel) makes it even easier for the initially trapped Ng atom to escape out of the cage of the [3${}_{n}$]CP molecule. The final result of geometry optimizations of the created endohedral complexes Ng@[3${}_{n}$]CP will be a resultant of both of these effects.

## 2. Results and Discussion

### 2.1. Structures of the Considered [3${}_{n}$]Cyclophanes

At the beginning, the investigated [3${}_{n}$]CPs will be presented, which were then used as host molecules to create model endohedral complexes Ng@[3${}_{n}$]CP. In the case of the parent molecule [3${}_{6}$]SP (i.e, [3${}_{6}$]CP), its structure will also be compared to the smaller analogue [2${}_{6}$]SP. The values of the most important structural parameters obtained for the [3${}_{n}$] (and additionally [2${}_{6}$]SP) cyclophanes are shown in Table 1, while the structures of individual cyclophanes will be briefly discussed in the following subsections.

#### 2.1.1. [3${}_{6}$](1,2,3,4,5,6)

**Figure 2:**
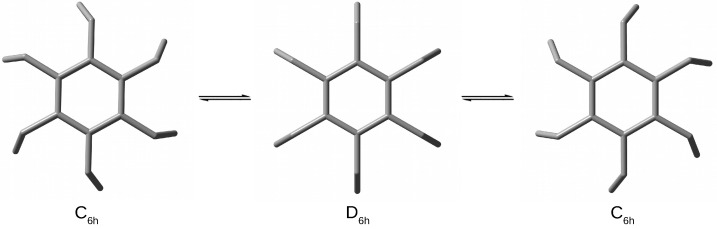
Top views of structures (hydrogen atoms have been removed for better visualization of the carbon backbones) of [3${}_{6}$](1,2,3,4,5,6)cyclophane conformers.

Compared to the recently considered [2${}_{6}$](1,2,3,4,5,6)SP [51,52], [3${}_{6}$](1,2,3,4,5,6)SP is characterized not only by a longer transannular distance (2.961 Å vs. only 2.654 Å), but also by significantly shorter C${}_{\mathrm{chain}}$-C${}_{\mathrm{chain}}$ (C${}_{c}$-C${}_{c}$ in Table 1) bonds (1.551 Å vs. as much as 1.592 Å) and a much greater value of the C${}_{\mathrm{ring}}$-C${}_{\mathrm{chain}}$-C${}_{\mathrm{chain}}$ ($\alpha_{C_{r}C_{c}C_{c}}$) angle (117.2${}^{\circ }$ vs. only 110.3${}^{\circ }$). It is also worth noting that the determined deviations of the C${}_{r}$-C${}_{c}$ bonds from the planes of the benzene rings are only 5.0${}^{\circ }$, while they are as much as 20.3${}^{\circ }$ in [2${}_{6}$](1,2,3,4,5,6)SP. Therefore, [3${}_{6}$](1,2,3,4,5,6)SP is characterized by a greater strain-free structure than [2${}_{6}$](1,2,3,4,5,6)SP. This in turn significantly affects the occurrence of the conformation phenomenon due to the flipping process of the six trimethylene bridges (Figure 2).

The structure of [3${}_{6}$](1,2,3,4,5,6)SP is often referred to as a pinwheel with six blades, and the possible conformations result from changes in their alignments. In the case of the C${}_{6h}$ form, the blades are bent in the same direction, resulting in the presence of a six-fold rotational axis (passing through the centers of both benzene rings), while their straightened alignments in the D${}_{6h}$ form additionally introduce vertical planes and two-fold axes. The stepwise interconversion among the respective conformations has been described previously in detail [70,71] and is therefore not the subject of this article. The calculations presented here are based on the fully optimized equilibrium form. Importantly, the full geometry optimization of [3${}_{6}$](1,2,3,4,5,6)SP has led to the conformer with C${}_{6h}$ symmetry, which is in full agreement with previous theoretical results [70,71]. The more symmetrical conformer with the $D_{6h}$ symmetry is characterized by as many as seven imaginary vibrational frequencies, which is also in full agreement with the previous theoretical result [71]. Moreover, this result has been confirmed by my calculations utilizing various DFT functionals and, additionally, the HF method. The D${}_{6h}$ form is energetically higher by about 36–50 kcal/mol depending on the method (42.7, HF; 36.4, B3LYP; 39.2, B3PW91; 39.6, TPSSh; 46.9, M06-L; 43.0, M06; 50.3, M06-HF; 49.9, M06-2X; 41.5, PBE0; 44.3, ωB97X-D). It should therefore be emphasized that the reported experimental structure with $D_{6h}$ symmetry results only from the known problem of the severe disorder of the conformers with $C_{6h}$ symmetry [72].

The analysis of the structural parameters presented in Table 1 suggests that the lowering of the symmetry from D${}_{6h}$ to C${}_{6h}$, associated with unidirectional deflections of the blades, is aimed at shortening the unfavorably long C-C bonds in the side chains, from as much as 1.567 Å to 1.551 Å. The bending of the blades also leads to a greater transannular distance ($d_{\pi \cdots \pi }$), from 2.919 Å in D${}_{6h}$ to 2.961 Å in C${}_{6h}$, and a significant reduction in the values of the side chain angles, i.e., $\alpha_{C_{r}C_{c}C_{c}}$ and $\alpha_{C_{c}C_{c}C_{c}}$, from 120.4${}^{\circ }$ to 117.2${}^{\circ }$ and from 124.7${}^{\circ }$ to 120.8${}^{\circ }$, respectively.

#### 2.1.2. [3${}_{5}$](1,2,3,4,5)

**Figure 3:**
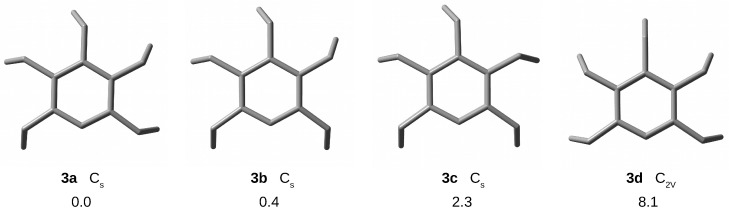
Top views of structures (hydrogen atoms have been removed for better visualization of the carbon backbones) and relative energies (in kcal/mol) of [3${}_{5}$](1,2,3,4,5)cyclophane conformers.

As a result of geometry optimizations of [3${}_{5}$](1,2,3,4,5)CP, I have obtained four conformers shown in Figure 3. The most stable of them turned out to be conformer **3a** characterized by unidirectional bending of the blades. Conformer **3b** is only slightly higher in energy; the relative energy is 0.4 kcal/mol. Even less energetically stable is the conformer **3c** (2.3 kcal/mol), while the **3d** conformer with C${}_{2V}$ symmetry is characterized by much higher energy, 8.1 kcal/mol. Most likely, this is due to the presence of one straightened blade.

It should be noted that Yasutake et al. [73] only reported conformers **3a**–**3c**, all with C${}_{s}$ symmetry, while they did not mention conformer **3d**, which is most likely due to its much higher energy. Therefore, this is the first report of this conformer. In my opinion, it is possible that there are even more conformers, but rather high-energy ones. As already mentioned, a thorough study of possible conformers of the discussed cyclophanes [3${}_{n}$], however, is not the subject of the presented study. Yasutake et al. [73] referred to the theoretical results of Shinmyozu et al. [67], where much less reliable calculations based on the MM3 force field suggested that conformers **3a** and **3c** are less stable than conformer **3b** by 0.7 and 1.3 kcal/mol, respectively. Yasutake et al. [73] also reported that form **3b** is the most stable in crystals.

The conformers of [3${}_{5}$](1,2,3,4,5)CP are characterized by a significant variation in the value of a given structural parameter (Table 1). For the transannular distance $d_{\pi \cdots \pi }$, the variation is 0.246 Å (**3a**), 0.329 Å (**3b**), 0.329 Å (**3c**), or 0.194 Å (**3d**). Importantly, the largest distance $d_{\pi \cdots \pi }$ characterizes the ring carbon atoms that are not bonded to each other by a trimethylene bridge. As can be seen, this distance is the largest in conformers **3b** (3.281 Å) and **3c** (3.279 Å). This suggests that escape of the initially trapped guest should be easier for these conformers than for **3a** and **3d**. Interestingly, conformers **3a**–**3c**, featuring C${}_{s}$ symmetry, have similar values of geometrical parameters ($d_{C_{r}C_{r}}$ = 1.39–1.41 Å, $d_{C_{r}C_{c}}$≈ 1.52 Å, $d_{C_{c}C_{c}}$ = 1.54–1.55 Å, $\alpha_{C_{r}C_{c}C_{c}}$ = 114–117${}^{\circ }$, $\alpha_{C_{c}C_{c}C_{c}}$ = 117–121${}^{\circ }$), while for the **3d** form, with C${}_{2V}$ symmetry, clear differences are visible. The C${}_{r}$C${}_{c}$ bonds are roughly equal in length (ca. 1.52 Å), and some of the trimethylene bridges are significantly more open (up to 121${}^{\circ }$ for $\alpha_{C_{r}C_{c}C_{c}}$ and up to 124${}^{\circ }$ for $\alpha_{C_{c}C_{c}C_{c}}$).

Compared to [3${}_{6}$](1,2,3,4,5,6)SP, it can be seen that the greatest structural effect resulting from the removal of one of the trimethylene bridges is a significant increase in the $d_{\pi \cdots \pi }$ distance between the carbon atoms not having such a bridge, i.e., at the escape channel. Descriptively, it can be said that [3${}_{5}$](1,2,3,4,5)CP creates a slightly upwardly tilted roof over the escape channel. In the study of the influence of Ng atom encapsulation inside the [3${}_{5}$](1,2,3,4,5)CP molecule, only its most energetically stable form **3a** was then considered.

#### 2.1.3. [3${}_{4}$](1,2,3,5)

**Figure 4:**
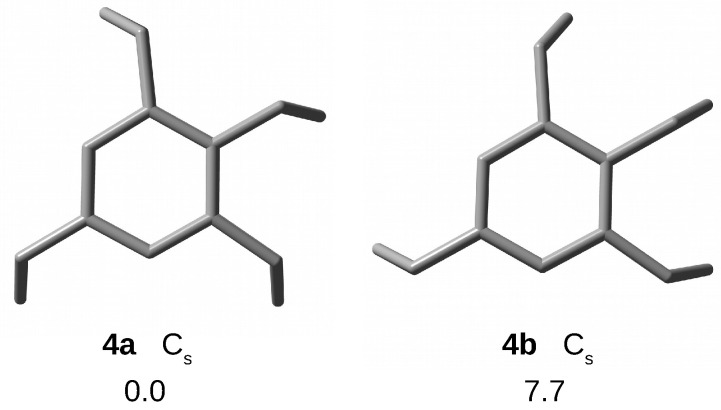
Top views of structures (hydrogen atoms have been removed for better visualization of the carbon backbones) and relative energies (in kcal/mol) of [3${}_{4}$](1,2,3,5)cyclophane conformers.

In the case of cyclophane [3${}_{4}$](1,2,3,5), Yasutake et al. [73] mentioned only one stable form with C${}_{s}$ symmetry (marked as **4a**). Instead, my calculations have led to two stable forms, **4a** and **4b**, both with C${}_{s}$ symmetry (Figure 4). However, form **4b** is much higher in energy (7.7 kcal/mol), which is most likely due to the presence of one straightened blade (similar to **3d**). Again, in further studies of endohedral complexes, only the more energetically stable conformer **4a** was considered.

Similarly to the more bridged [3${}_{5}$](1,2,3,4,5)CP, also [3${}_{4}$](1,2,3,5)CP is characterized by a certain dispersion of the values of the same structural parameters (Table 1). Compared to **3a**, **4a** shows somewhat larger $d_{\pi \cdots \pi }$ distances, with the largest value of 3.228 Å for unbridged ring carbon atoms. Thus, again, the benzene rings form characteristic small upward bends in these places. Interestingly, the values of the remaining geometric parameters in form **4a** are practically identical to those in **3a**. Only in the case of the angle $\alpha_{C_{c}C_{c}C_{c}}$, a much greater differentiation of values is noticeable (116.8–120.3${}^{\circ }$).

#### 2.1.4. [3${}_{4}$](1,2,4,5)

**Figure 5:**
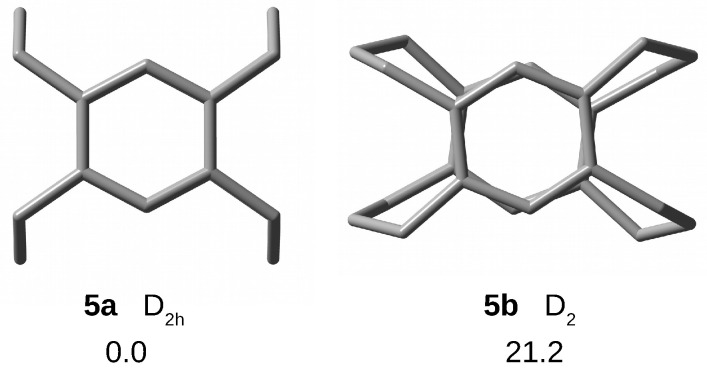
Top views of structures (hydrogen atoms have been removed for better visualization of the carbon backbones) and relative energies (in kcal/mol) of [3${}_{4}$](1,2,4,5)cyclophane conformers.

In the case of [3${}_{4}$](1,2,4,5)CP, two conformers, **5a** with high D${}_{2h}$ symmetry and **5b** with D${}_{2}$ symmetry, have been obtained (Figure 5). As can be seen from Figure 5, conformer **5b** is characterized by twisting of the trimethylene bridges and benzene rings, resulting in a loss of the horizontal plane. This form is by as much as 21.2 kcal/mol higher in energy than the more stable form **5a**, which is probably why it was not mentioned at all in ref. [73]. Therefore, again, this is the first report of conformer **5b**. The significantly higher energy of the twisted form **5b** shows that both the benzene rings and the trimethylene bridges prefer to remain untwisted and indeed untwisted structures are characteristic of simpler cyclophanes [50].

Compared to **4a** (and **3a** and **2**), **5a** is characterized by slightly larger $d_{\pi \cdots \pi }$ spacing, with the largest distance (3.247 Å) again being for the unchained carbon atoms. As a consequence, both benzene rings have a distinct boat structure, making it easier for the initially trapped guest to escape beyond the cage structure of the cyclophane. Apart from $d_{C_{r}C_{r}}$ (1.392–1.400 Å), the values of other geometrical parameters of **5a** are close to the lower values for **4a** (Table 1).

#### 2.1.5. [3${}_{3}$](1,3,5)

**Figure 6:**
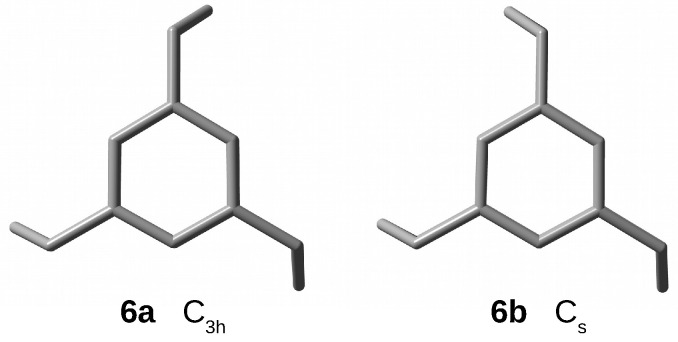
Top views of structures (hydrogen atoms have been removed for better visualization of the carbon backbones) of [3${}_{3}$](1,3,5)cyclophane conformers.

Referring to the VT ${}^{1}$H NMR study in CD${}_{2}$Cl${}_{2}$ by Meno et al. [66], Yasutake et al. [73] reported two forms of [3${}_{3}$](1,3,5)CP, of which the one with C${}_{s}$ symmetry (**6b**) was the most stable, while the one with C${}_{3h}$ symmetry (**6a**) was energetically higher by only 0.4 kcal/mol (Figure 6). However, the relative energies can strongly depend on the solvent. Geometry optimizations at the ωB97X-D/6-311++G(d,p) level of theory have given practically the same total energies of both conformers (therefore, both were then used to form endohedral complexes Ng@[3${}_{3}$](1,3,5)CP).

Compared to **6a**, the conformer **6b** is characterized by a slightly greater variation in the values of the same geometrical parameters, especially the $d_{\pi \cdots \pi }$ distance (Table 1). In the former form, this variation amounts to 0.050 Å, while in the latter it is as much as 0.108 Å. In both cases, again, the longest $d_{\pi \cdots \pi }$ distance concerns the unchained carbon atoms of the benzene rings and it is slightly shorter (3.168 and 3.212 Å in **6a** and **6b**, respectively) than in cyclophanes **5a** and **4a**, having four trimethylene bridges. Of course, [3${}_{3}$](1,3,5)CP contains the largest number (three) of single-carbon windows.

### 2.2. The Ng@[3${}_{n}$]cyclophane Endohedral Complexes

The fully optimized structures of the previously discussed cyclophanes were then used to create the initial model structures of the Ng@[3${}_{n}$]CP endohedral complexes. Then, geometry optimizations of the complexes created in this way were carried out. Due to the unique structure of the [3${}_{6}$](1,2,3,4,5,6)cyclophane, i.e., [3${}_{6}$]superphane or compactly [3${}_{6}$]SP, its endohedral complexes will be discussed first. The obtained results will also be compared to those previously obtained for endohedral complexes of [2${}_{6}$]SP [52]. Then, in the next subsection, the endohedral complexes of the remaining cyclophanes under consideration (i.e., with 3 ≤ *n* ≤ 5) will be discussed.

#### 2.2.1. Endohedral Complexes of the [2${}_{6}$] and [3${}_{6}$] Superphanes

**Figure 7:**
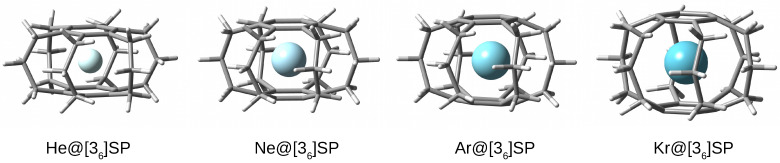
Structures of the Ng@[3${}_{6}$]SP endohedral complexes (Ng = He, Ne, Ar, Kr; SP = superphane).

Since superphanes have a full set of six bridges connecting both benzene rings (see Figure 1 and Figure 2), their structure is the most perfect cage. It is, therefore, not an unexpected result that the full geometry optimizations of the designed Ng@SP endohedral complexes did not lead to the escape of the trapped Ng atom from the superphane cage (however, such an escape is possible for the largest Kr atom and [2${}_{6}$]SP when some less reliable levels of theory are used; for details see ref. [51]). Thus, each of the Ng atoms remained inside the superphane molecule, thus creating an endohedral complex (Figure 7). The characteristics of these complexes are given in Table 2.

As already mentioned in the Introduction section, it was shown earlier [52] that the encapsulation of the Ng atom inside the superphane [2${}_{6}$] leads to significant changes in the values of geometric parameters, indicating the “swelling” of the [2${}_{6}$]SP molecule. This effect becomes more and more pronounced as the radius of the Ng atom increases, i.e., into the He→Ne→Ar→Kr series. This swelling is expressed by a significant increase in the length of the C-C bonds and the value of the angle $\alpha_{C_{r}C_{c}C_{c}}$ (larger opening), but above all a significant increase in the transannular distance $d_{\pi \cdots \pi }$ (Table 2). Of course, the swelling effect of [2${}_{6}$]SP is greatest in the case of the largest Kr atom. It is worth noting that the presence of this atom inside the [2${}_{6}$]SP molecule increases the distance between the benzene rings by as much as about 1.050 Å (from 2.654 Å up to ca. 3.704 Å), i.e., by about 40%. Moreover, the already long C${}_{c}$C${}_{c}$ bond (e.g., in ethane, it is ‘only’ 1.526 Å) extends from 1.592 Å up to 1.751–1.754 Å, making it one of the longest C-C bonds ever reported (!) [78,79,80,81,82,83,84,85].

Exactly the same pattern of structural changes, i.e., “swelling”, occurs in the case of Ng@[3${}_{6}$]SP. However, the observed changes in the C${}_{r}$C${}_{r}$, C${}_{r}$C${}_{c}$ and C${}_{c}$C${}_{c}$ bonds as well as the $\alpha_{C_{r}C_{c}C_{c}}$ angle are much smaller. For example, the C${}_{c}$C${}_{c}$ chain bond, in the case of insertion of a Kr atom, is extended to ‘only’ about 1.620 Å, i.e., by only 4% compared to 10% for the Kr@[2${}_{6}$]SP complex. Nevertheless, as shown by the last column in Table 2, the angle $\alpha_{C_{c}C_{c}C_{c}}$, i.e., between chain carbon atoms, undergoes very large changes. Inserting a He atom increases this angle by 3${}^{\circ }$, a Ne atom by 8${}^{\circ }$, while an Ar or Kr atom by as much as 14.5${}^{\circ }$ and 17${}^{\circ }$, respectively, which is 12% and 14% of the value for the empty [3${}_{6}$]SP molecule. However, the largest, and in some cases even surprisingly huge, changes have been obtained for the $d_{\pi \cdots \pi }$ distance. Namely, the insertion of a He atom inside [3${}_{6}$]SP increases the $d_{\pi \cdots \pi }$ distance by 0.233 Å (from 2.961 Å to 3.194 Å), i.e., by 7.9%. Replacing the He atom with a Ne atom leads to a further increase in the $d_{\pi \cdots \pi }$ distance by another 0.366 Å, i.e., by 11.5%, which in relation to the empty [3${}_{6}$]SP gives an increase of 0.599 Å (20.2%). However, the most spectacular $d_{\pi \cdots \pi }$ changes occur in the presence of either Ar or Kr. In the former case, the $d_{\pi \cdots \pi }$ distance is increased by 1.207 Å (i.e., 40.8% relative to [3${}_{6}$]SP), while in the latter one by as much as 1.288 Å, which is as much as 43.5% of the $d_{\pi \cdots \pi }$ value in the empty [3${}_{6}$]SP. Such a huge increase in the transannular distance (clearly visible in Figure 7) shows the high structural flexibility of the tested cyclophanes and their value in the study of encapsulation effects.

**Figure 8:**
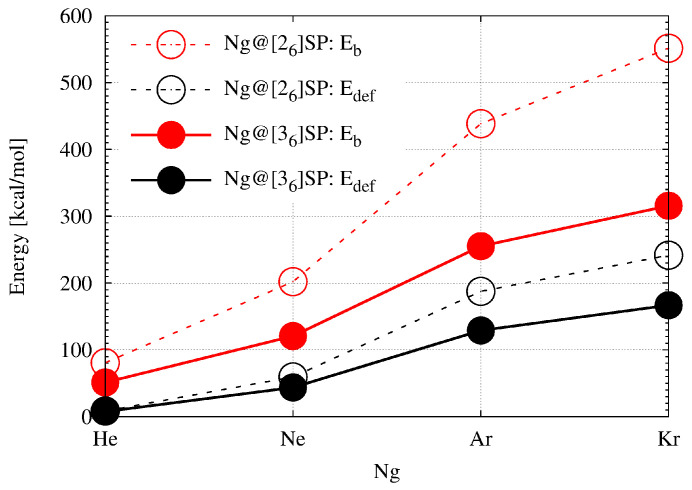
Dependence of the binding ($E_{b}$) and deformation ($E_{\mathrm{def}}$) energy on the noble gas atom (Ng = He, Ne, Ar, Kr) in the endohedral Ng@[2${}_{6}$]SP and Ng@[3${}_{6}$]SP (SP = superphane) complexes.

It is obvious that the “swelling effect” of the superphane molecule is energetically unfavorable, which can be seen in the rather high values of the deformation energy ($E_{\mathrm{def}}$ in Table 2). Although the insertion of a He atom yields only 7.7 kcal/mol, the deformation energy of the superphane [3${}_{6}$] increases rapidly, from about 44 kcal/mol for Ne through 129 kcal/mol for Ar up to 167 kcal/mol for Kr. It is expected that the deformation energy should increase with decreasing cage size, and indeed the deformation energies for [2${}_{6}$]SP with encapsulated He, Ne, Ar, or Kr atoms are 8.5, 59.5, ca. 188, and ca. 242 kcal/mol (for a comparison of the values obtained for Ng@[3${}_{6}$]SP and Ng@[2${}_{6}$]SP see also Figure 8). For even smaller adamantane, the deformation energy obtained for the He@adamantane complex was as high as 15.3 kcal/mol [12,13]. It is worth noting that despite the larger cavity in [3${}_{6}$]SP than in [2${}_{6}$]SP, for a given Ng atom, the percentage contribution of the deformation energy to the binding energy is higher in the former of these superphanes and exceeds 50% for Ar and Kr atoms.

As mentioned in the Introduction section, according to Bader’s QTAIM, the simultaneous presence of a bond path (BP) and its associated critical point (BCP) between any two atoms is both a necessary and sufficient condition for the atoms to be bonded to one another [1]. As the bonded atoms should give a stabilizing contribution, the computed binding energy ($E_{b}$ in Table 2) is the key quantity in this article. Most importantly, as can be seen from the second column of Table 2, the obtained $E_{b}$ values are positive for all Ng@[3${}_{6}$]SP (and Ng@[2${}_{6}$]SP) [52]) endohedral complexes. This result indicates that the Ng⋯SP intra-cage interactions are non-bonding, i.e., they are destabilizing. Of course, as might have been expected, the non-bonding effect increases rapidly as the size of the Ng atom increases (He→Ne→Ar→Kr). It is also clearly larger for the smaller superphane [2${}_{6}$] (see Table 2 and Figure 8). Large positive $E_{b}$ values together with large energetically unfavorable deformations of the superphane structure show that encapsulation is certainly an energetically unfavorable process.

#### 2.2.2. Endohedral Complexes of the [2${}_{n}$] and [3${}_{n}$] One-Carbon Window Cyclophanes

**Figure 9:**
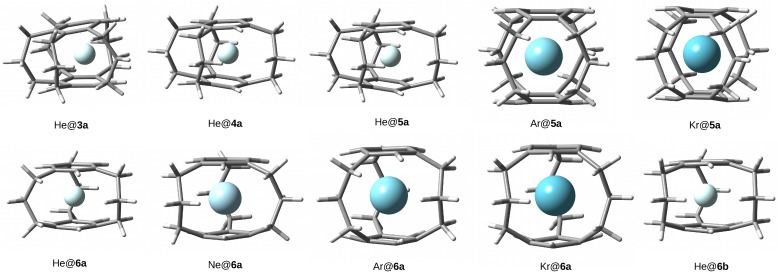
Structures of the Ng@[3${}_{n}$]CP endohedral complexes (Ng = He, Ne, Ar, Kr; CP = cyclophane).

Whether a guest trapped in the host’s cage prefers to stay there or, on the contrary, prefers to escape from it, can be easily verified by performing geometry optimizations for endohedral guest@host complexes involving a host molecule possessing at least one escape channel. For this purpose, cyclophanes with a reduced number of trimethylene chains (3 ≤*n*≤ 5) were utilized and then their Ng@[3${}_{n}$]CP complexes were built. However, in order to preserve the cage structure of the cyclophane molecule and at the same time not to facilitate the escape of the Ng atom too much, only those [3${}_{n}$]CPs with one-carbon escape channels (additionally, only their most stable forms) have been investigated. These were cyclophanes **3a** (Figure 3), **4a** (Figure 4), **5a** (Figure 5), **6a**, and **6b** (Figure 6). Geometry optimizations of their endohedral complexes Ng@[3${}_{n}$]CP in most cases have led to the escape of the initially trapped Ng atom from the cyclophane cage with the formation of the CP⋯Ng exohedral complex (these cases will be discussed later). However, in ten cases, the trapped Ng atoms have remained inside the cyclophane molecules, thus forming the Ng@[3${}_{n}$]CP endohedral complexes. The structures of these complexes are shown in Figure 9, while the binding and deformation energies as well as some selected structural parameters are shown in Table 3.

Firstly, it should be clearly noted that the Ng atom remaining inside the cyclophane molecule does not mean a stabilizing interaction between this atom and the interior of the cyclophane cage. Quite the opposite, this interaction is non-bonding (repulsive), which is clearly indicated by the positive values of the binding energy obtained in each of these cases. Of course, the formation of the endohedral complex leads to strong disturbances in the structure of the host molecule, which in the case of the considered cyclophanes is manifested by structural ‘swelling’ already discussed in the example of superphanes (see Section 2.2.1). This is best seen in the case of cyclophane **6a** (i.e., form **6a** of [3${}_{3}$](1,3,5)CP; Figure 6), which forms an endohedral complex with each of the four Ng atoms. For example, the maximum $d_{\pi \cdots \pi }$ distance increases from 3.168 Å in **6a** to 3.540 Å in He@**6a**, then to 4.039 Å in Ne@**6a** and 4.563 Å in Ar@**6a**, and up to 4.722 Å in Kr@**6a**. In the last case, this is an increase of almost 50%. The systematic and rapid increase in the transannular distance for these complexes is clearly visible in the bottom row of Figure 9.

Cyclophanes **3a** and **4a**, featuring similar maximum $d_{\pi \cdots \pi }$ values (3.204 and 3.228 Å, respectively), retain only the smallest He atom, while the larger Ne, Ar, and Kr atoms escape from their interiors, forming exohedral complexes. It is interesting to compare the number of endohedral complexes obtained for **4a** and **5a**, i.e., cyclophanes having the same number (four) of trimethylene bridges, but differing in their location (see Figure 4 and Figure 5). The former forms an endohedral complex only with the smallest He atom, while the latter with He, Ar, and Kr. This can most likely be explained by the greater transannular distance in the latter case, which indicates a larger trapping cavity, and thus, slightly weaker Ng⋯C repulsion inside the cyclophane. It is quite surprising that the Ne atom is removed from the cage. Apparently, in this case, the internal repulsive forces outweighed the possibility of trapping the atom. A much less obvious situation concerns forms **6a** and **6b**, as both forms have the same locations of trimethylene bridges (i.e., 1,3,5) and differ only in the opposite orientation of one of the blades (see Figure 6). The structural difference is therefore more subtle. However, it seems that the much larger maximum $d_{\pi \cdots \pi }$ distance in the **6b** form (3.212 Å vs. 3.168 Å in **6a**) allows the Ne, Ar, and Kr atoms to escape.

**Figure 10:**
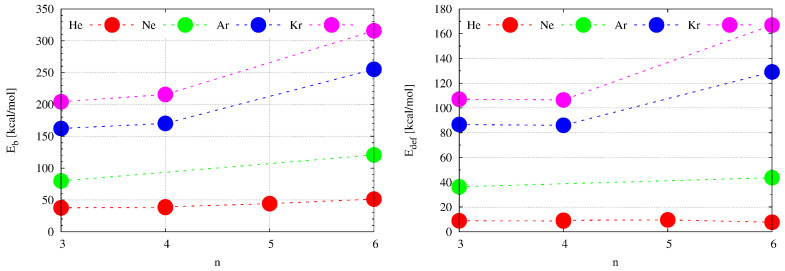
Dependence of binding energy (**left**) and deformation energy (**right**) on the number of trimethylene side chains, *n*, in the Ng@[3${}_{n}$]CP (CP = cyclophane) endohedral complexes.

It is interesting to compare the encapsulation-induced structural changes in the cyclophane complexes presented in Table 3 with the structural changes occurring in the superphane complexes shown in Table 2. As already mentioned in Section 2.2.1, the insertion of He, Ne, Ar, or Kr inside [3${}_{6}$]SP leads to an increase in the average $d_{\pi \cdots \pi }$ distance by approx. 8%, 20%, 37%, and 43%, respectively. A similar operation for the structurally looser **6a** leads to increases of 11%, 25%, 42%, and 47%, respectively. Interestingly, the percentages are similar even for the more cagey cyclophanes **3a** and **5a**. Insertion of He into the former gives 11.6%, while insertion of He, Ar, or Kr into the latter gives 11%, 41%, and 46%, respectively. Therefore, it seems that the percentage increase in the $d_{\pi \cdots \pi }$ distance depends mainly on the type of the trapped Ng atom, and to a much lesser extent on the one-carbon window cyclophane. This result also translates into deformation energies which, for a given Ng atom, are almost independent of the type of cyclophane: ca. 9 kcal/mol for He, ca. 86 kcal/mol for Ar, and 107 kcal/mol for Kr. However, the binding energy depends much more clearly on the structure of the cyclophane molecule (Figure 10).

### 2.3. Bader’s Bond Path Does Not Necessarily Indicate Stabilizing Interaction

#### 2.3.1. QTAIM-Based Study

**Figure 11:**
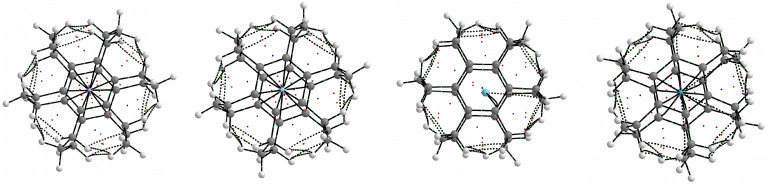
Molecular graphs of the Ng@[3${}_{6}$]SP (Ng = He, Ne, Ar, Kr; SP = superphane) endohedral complexes.

The results presented in Table 2 and Table 3 show conclusively that in the cases of all of the obtained endohedral complexes, the binding energies have positive values, and therefore, the Ng⋯CP/SP interactions inside the cage are certainly non-bonding, i.e., repulsive. This conclusion is in full agreement with the previous results [12,13,15,16,17,51,52]. For example, Moran et al. [17] stated that “exohedral binding is preferred to endohedral encapsulation without exception”. However, it will be shown that the trapped Ng atom quite willingly creates many bond paths to carbon atoms, and not only the closest ones, and their total number is very sensitive to even small shifts of the Ng atom from the center of the cage. Molecular graphs of the resulting Ng@[3${}_{6}$]SP endohedral complexes are shown in Figure 11.

As can be seen, He@[3${}_{6}$]SP and Ne@[3${}_{6}$]SP have 12 bond paths of the Ng⋯C${}_{r}$ type, which results from the high symmetry (C${}_{6h}$) of these complexes. However, the Ar@[3${}_{6}$]SP complex has only two such bond paths, which results from a slight shift of the Ar atom from the center of the superphane molecule. The most surprising result, however, is that the Ar atom also creates one bond path to the distant (because the middle) carbon atom of one of the six trimethylene chains (i.e., Ar⋯C${}_{c}$). The Kr atom in the Kr@[3${}_{6}$]SP complex forms 12 ‘regular’ Kr⋯C${}_{r}$ BPs, but also, additionally, 4 BPs of the Kr⋯C${}_{c}$ type. The number four and not six results from breaking the rotational symmetry as a result of a negligible shift of the Kr atom from the center.

**Figure 12:**
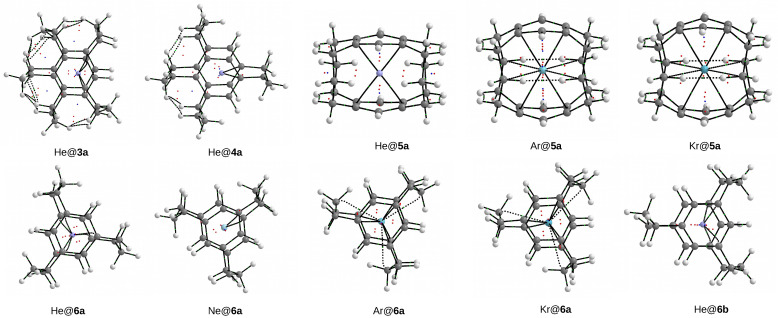
Molecular graphs of the Ng@[3${}_{n}$]CP (Ng = He, Ne, Ar, Kr; CP = cyclophane) endohedral complexes.

Molecular graphs of the obtained Ng@[3${}_{n}$]CP endohedral complexes (Figure 9) are shown in Figure 12. A thorough analysis of these molecular graphs leads to the conclusion that the He atom (and Ne) creates only bond paths to the $C_{r}$ ring carbon atoms, with the number of bond paths (four in He@**3a**, two in He@**4a**, six in He@**6a**, and four in He@**6b**) depending on the position of this atom relative to them, or to the bond critical point of the $C_{r}$C${}_{r}$ bond (four in He@**5a**), which can be seen as He⋯π bond paths. On the contrary, the much larger Ar and Kr atoms, in addition to the Ng⋯C${}_{r}$ and Ng⋯π bond paths, whose number (four in Ar@**5a** and Kr@**5a**, six in Ar@**6a** and Kr@**6a**) again depends on the position of these atoms relative to the C${}_{r}$ atoms (and the center of the cage), are also more likely to form additional bond paths to the middle carbon atoms of the trimethylene side chains (four in Ar@**5a** and Kr@**5a**, three in Ar@**6a** and Kr@**6a**). This may seem to be surprising considering the considerable distance Ng⋯C${}_{c}^{\mathrm{middle}}$, however, it is known [34] that the presence or absence of a bond path does not necessarily depend on the interatomic distance.

**Figure 13:**
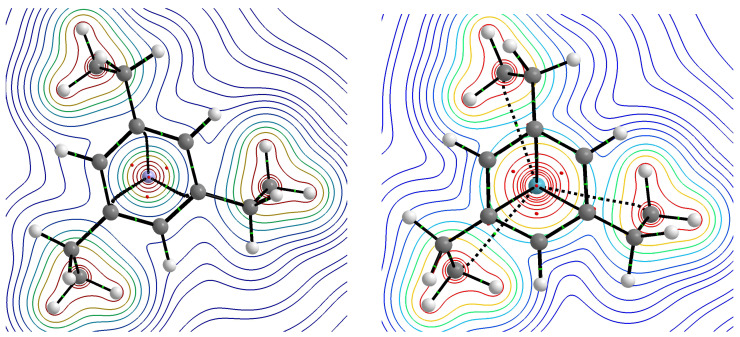
Contours of the electron isodensity in the plane of the Ng atom and the middle atoms of the trimethylene side chains of the He@**6a** (**left**) and Kr@**6a** (**right**) endohedral complexes.

To understand the reason for the presence of Ng⋯C${}_{c}$ bond paths in argon and krypton complexes, it is enough to compare the contours of electron isodensity determined, for example, for the He@**6a** and Kr@**6a** complexes. Figure 13 shows such contours obtained for the plane of the Ng atom and the middle atoms of the trimethylene side chains. The small size of the He atom means that its electron density distribution ‘does not find’ the electron density distributions of the -CH${}_{2}$- groups. On the other hand, the larger size of the Kr atom is obviously associated with a larger distribution of its electron density, which therefore easily ‘finds’ the electron density distributions of -CH${}_{2}$- groups. By merging with them, three Ng⋯C${}_{c}$ bond paths are formed (dashed lines in the right subfigure of Figure 13). In a similar way, i.e., by looking at the electron isodensity contours, the presence of different types (e.g., Cl⋯F, Cl⋯Cl) of counterintuitive bond paths in different dimers was explained [32,33,34].

At the end of this subsection, it should be emphasized once again that due to the positive values of binding energies obtained without exception for the Ng⋯SP/CP interactions in all the endohedral complexes investigated, the obtained results show that the presence of a BP does not necessarily indicate a stabilizing interaction. Yet another argument in support of this statement is provided in the next subsection. By the way, it is worth mentioning that the molecular graph itself can depend on the level of theory used [33,34,35,51,86].

#### 2.3.2. Spontaneous Escape of the Ng Atom from the Cage—Yet Another Proof

It has already been discussed (Section 2.2) that in 14 cases out of 24, the trapped Ng atom remained inside the cyclophane (or superphane) cage, forming the corresponding Ng@[3${}_{n}$]CP (or Ng@[3${}_{6}$]SP) endohedral complexes. However, in these cases the $E_{b}$ values are always positive (Table 2 and Table 3), proving the nonbonding nature of the Ng⋯SP/CP interaction in the cage. Contrarily, in the remaining 10 cases, the full geometry optimizations of the initially constructed Ng@[3${}_{n}$]CP endohedral complexes have led to the removal of the trapped Ng atom from the cage and the formation of exohedral complexes. Importantly, during the geometry optimization of the endohedral complex, the escape of the Ng atom from the interior of the cyclophane cage occurs spontaneously.

**Figure 14:**
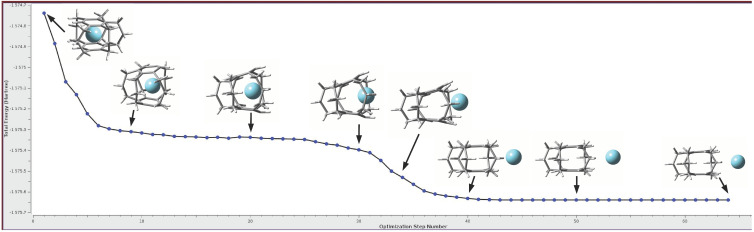
Change of total energy and structure of Ar⋯**3a** complex. On the left, the endohedral complex Ar@**3a** is visible, then, the escape of the Ar atom from its interior leads to the formation of the exohedral complex **3a**⋯Ar (right). After the escape of the Ar atom, the structure of the cyclophane molecule **3a** is recreated.

Interestingly, after the removal of the Ng atom, the structure of the cyclophane is restored, so that deformation energies are zero in all cases. Therefore, it is worth recalling here that in the case of smaller [2${}_{n}$]cyclophanes, their structures after the removal of the Ng atom were, in several cases, significantly changed or even destroyed [52]. Of course, due to the fact that one of the interacting subsystems is the Ng atom, the binding energies are small, ca. −1 kcal/mol for Ne, ca. −1.3 kcal/mol for Ar, and ca. −2 kcal/mol for Kr. Figure 14 shows changes in the total energy and structure of the Ar@**3a** complex during its geometry optimization. It also represents well other cases where the geometry optimization of the initially constructed endohedral complex has led to the removal of the Ng atom with the formation of a weak exohedral complex.

In the case shown in Figure 14, even a slight movement of the Ar atom from the center of the **3a** cyclophane cage together with a change in the structure gives an energy gain of ca. −360 kcal/mol (step 9), moving this atom to the exit channel gives a total energy gain of about −376 kcal/mol (step 20), entering the Ar atom into the escape window area gives a total reduction in the total energy of −413 kcal/mol (step 30). After this stage, there is a strongly exothermic stage of the Ar atom getting out of the cyclophane area, and then a very flat area of the potential curve related to the formation of the **3a**⋯Ar exohedral complex. The entire process of removing the Ar atom from the interior of the cyclophane **3a** together with the formation of the exohedral complex **3a**⋯Ar is strongly exothermic (−565 kcal/mol).

The process of spontaneous escape of the Ar atom from the inside of the cyclophane, illustrated in Figure 14, taking place during the optimization of the geometry of the endohedral Ar@**3a** complex, clearly shows that the initially trapped Ar atom does not want to be inside the cage structure of the cyclophane. This is of course related to the unfavorable energy effect of the encapsulation process. Recalling the result shown earlier (Section 2.3.1), that Ar, like the rest of the Ng atoms, willingly forms bond paths to the carbon atoms of the cage, it becomes clear that these paths do not result from intra-cage stabilization.

#### 2.3.3. The NCI-Based Studies

**Figure 15:**
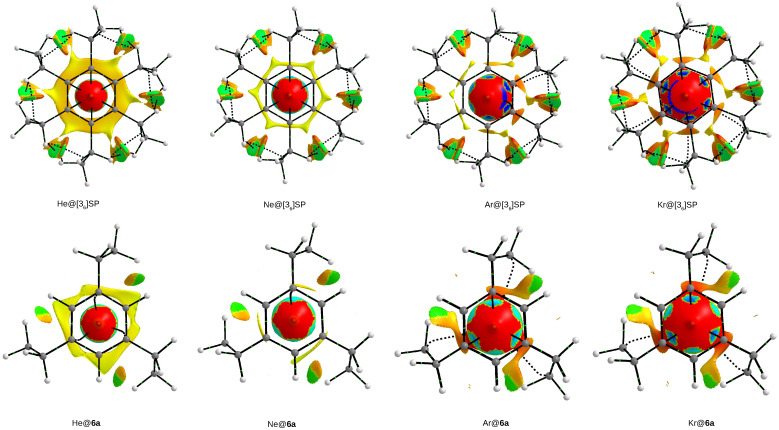
Top views of NCI-based *s*-isosurfaces (*s* = 0.5 a.u. for the Ng@[3${}_{6}$]SP and Ng@**6a** (Ng = He, Ne, Ar, Kr; SP = superphane) endohedral complexes. Colors are coded according to a common sgn($\lambda_{2}$)ρ scale (in a.u.): −0.040—blue, −0.025—cyan, −0.010—green, 0.005—yellow, and 0.020—red. A cutoff of 0.050 a.u. was used for the electron density.

The nature of the Ng⋯C interactions inside the Ng@[3${}_{6}$]SP and Ng@[3${}_{n}$]CP endohedral complexes is now investigated using NCI, i.e., the noncovalent interaction index [87,88]. Particularly interesting were the observations of changes in the areas of weak attraction and weak repulsion with an increase in the size of the Ng atom, i.e., into the He→Ne→Ar→Kr series, and changes in these areas (i.e., *s*-isosurfaces) after reducing the number of trimethylene side chains. Due to the fact that superphane and the cyclophane **6a** were the only ones to form endohedral complexes with all Ng atoms (Figure 7 and Figure 9), the set of *s*-isosurfaces obtained for them is shown in Figure 15.

First, let us note that the NCI-based *s*-isosurfaces are dominated by red and yellow colors, which, according to the adopted coding, indicates repulsive interactions. In all cases, regardless of the type of Ng atom or cyclophane, this repulsive area dominates along the six-fold rotational axis of the benzene rings, creating a characteristic spindle-like shape. However, this area clearly depends on the type of the Ng atom, expressing, of course, a large dependence on the size of this atom. In addition, in the case of Ne and especially the larger Ar and Kr atoms, six small areas of weak attraction (blue and cyan) resulting from the Ng⋯C${}_{r}$ contacts are also visible in the mentioned large spindle-shaped repulsion region (arc-shaped areas in complexes Ar@[3${}_{6}$]SP and Kr@[3${}_{6}$]SP result from a slight asymmetry of the position of either the Ar or Kr atom relative to the center of the rings). It should be emphasized that these interactions are attractive according to the NCI-based interpretation, which, however, does not refer to their energetics. As clearly seen, these six (and another six on the other side) spots are the only areas of weak attraction inside the cage structure of a superphane or cyclophane.

What seems to be characteristic of He, its He@[3${}_{6}$]SP and He@[3${}_{6}$]**6a** complexes form a fairly wide disc region of very weak repulsion (yellow) extending beyond the inter-ring contact. Its greater spatial prolixity in the former case indicates greater compression inside the superphane than in the cyclophane **6a**. In the case of [3${}_{6}$]SP complexes, this area narrows in the case of Ne to fragment in complexes with Ar and Kr.

While discussing the NCI-based *s*-isosurfaces shown in Figure 15, it is also worth mentioning the confluent areas of weak repulsion and very weak attraction (green), which seem to be quite characteristic for the peripheral C-H⋯H-C interactions. According to the NCI, this result indicates weakly attractive interactions of this type. Most likely, the presence of such areas was first described by Johnson et al. in branched octane in their 2010 paper on the NCI method [87], and recently by me in the so-called ‘iron maiden’ molecules [36,37] and between ethylene groups in ZnEt${}_{2}$ in complexes with carbenes [89].

## 3. Methodology

The initial phase of calculations consisted of geometry optimizations of [3${}_{6}$](1,2,3,4,5,6) superphane and its derivatives with a reduced number of trimethylene chains (3 ≤ *n* ≤ 5), yet featuring at most one-carbon windows in the skeleton ([3${}_{n}$]cyclophanes: [3${}_{5}$](1,2,3,4,5), [3${}_{4}$](1,2,4,5), [3${}_{4}$](1,2,3,5), and [3${}_{3}$](1,3,5)). The choice of only the cyclophanes with one-carbon windows was made to allow the initially trapped Ng atom to escape from the cyclophane while still ensuring its cage structure. Increasing the size of the carbon window would lead to a more open shell-like cyclophane structure [52]. Then, the fully optimized structures of these cyclophanes were utilized to construct initial geometries of Ng@[3${}_{6}$]SP and Ng@[3${}_{n}$]CP (Ng = He, Ne, Ar, Kr) endohedral complexes, which were then re-optimized. Geometry optimizations were performed at the ωB97X-D/6-311++G(d,p) level of theory, i.e., within the ωB97X-D exchange-correlation functional [90] of density functional theory [91,92] and the 6-311++G(d,p) basis set [93,94]. As shown [95], the ωB97X-D functional is one of the best among 200 tested for general purposes, including intermolecular interactions. Moreover, this functional reproduced the crystallographic structure of [2${}_{6}$]SP [47] well [51]. On the other hand, the 6-311++G(p,d) basis set contains diffuse functions on all atoms, which are necessary for a reliable description of electrons located at a greater distance from atomic nuclei, as is the case with such interactions. The frequency analysis confirmed that true minima were obtained on the potential energy surface (no imaginary frequencies). It should be noted that in some cases several conformers of a given cyclophane were obtained (see the Results and Discussion section). Both geometry optimizations and frequency calculations were performed using the Gaussian 16 package [96]. Graphical representation of the molecules and the total energy curve were obtained with the GaussView 6 program [97]. The Cartesian coordinates of the considered systems can be found in Supplementary Materials.

For the obtained complexes (endohedral or exohedral), the binding energy ($E_{b}$) between the Ng atom and the cyclophane molecule was calculated:

(1) $$E_{b}=E\left(\mathrm{complex}\right)-E\left(\mathrm{cyclophane}\right)-E\left(\mathrm{Ng}\right)$$

where *E*(complex), *E*(cyclophane), and *E*(Ng) are the total energies of the entities shown in the parentheses. In the case of endohedral complexes, $E_{b}$ can also be seen as the inclusion energy [17]. Crucially, a negative value of binding (inclusion) energy indicates the stabilizing (bonding) nature of the Ng⋯cyclophane interaction, while a positive value means that such an interaction is non-bonding (repulsive) [98]. The deformation (distortion) energy ($E_{\mathrm{def}}$) of the cyclophane molecule was simply calculated as the difference between the total energies of the cyclophanes within their complex and equilibrium geometries:

(2) $$E_{\mathrm{def}}=E\left({\mathrm{cyclophane}}^{★}\right)-E\left(\mathrm{cyclophane}\right)>0$$

The deformation energy, which obviously is positive, can be understood as the energetic penalty paid by the cyclophane molecule while changing its structure from the optimal, i.e., equilibrium one, to the one in the complex.

In order to obtain non-local insight into the nature of the Ng⋯C interactions within the structures of the endohedral Ng@[3${}_{6}$]SP and Ng@[3${}_{n}$]CP complexes, the noncovalent interaction index method (NCI) [87,88] was used. This method is based on the reduced electron density gradient ($s=1/\left(2{\left(3\pi^{2}\right)}^{1/3}\right)\left|\nabla \rho \right|/\rho^{4/3}$) and $sgn(\lambda_{2})\rho$, i.e., the electron density multiplied by the sign of the second eigenvalue of the electron density Hessian matrix ($\lambda_{2}$). As a consequence, NCI allows for displaying individual weak interactions as certain regions of real space rather than as local features of a BCP corresponding to a pairwise interatomic contact. Most importantly, these interactions can be easily and visually (by using different colors) separated into attractive (if $\lambda_{2}<0$) and repulsive (if $\lambda_{2}>0$) [87,88]. Molecular graphs and NCI-based *s*-isosurfaces were obtained utilizing the AIMAll program [99].

## 4. Conclusions

The main ‘working’ achievement of Bader’s quantum theory of atoms in molecules (QTAIM) was the deriving of a strong theorem, according to which the simultaneous presence of a bond path (BP) and the associated bond critical point (BCP) between any two atoms is both a necessary and sufficient condition for the atoms to be bonded to one another. It is obvious that the atoms ‘bonded to one another’ should provide a stabilizing energetic effect. However, there are many bond paths (so-called counterintuitive bond paths) for which the stabilizing effect of an interaction is at least questionable. An excellent example are endohedral complexes, in which there is often a surprisingly large number of counterintuitive bond paths between the trapped guest (e.g., atom) and the atoms (usually carbons) of the cage structure of the host.

Until recently, methods to demonstrate the repulsive nature of the guest⋯host interactions were quite limited due to the solid backbone structure of the host molecule, which prevented the trapped entity from spontaneously escaping out of the host molecule. In order to show the significant effect of an encapsulation on the structure of the host molecule, a very flexible [3${}_{6}$](1,2,3,4,5,6)SP (SP = superphane) molecule with two benzene rings connected by six trimethylene bridges has been used. It has been shown that the encapsulation of a noble gas atom (Ng) inside [3${}_{6}$]SP, with the formation of an endohedral complex Ng@[3${}_{6}$]SP, leads to ‘swelling’ of the superphane structure manifested primarily by a significant increase in the transannular distance π⋯π. Moreover, the obtained positive binding energy values show that the Ng⋯SP interaction inside the SP cage is in fact destabilizing.

The destabilizing nature of the Ng⋯host interactions has been shown even better by performing geometry optimizations of model endohedral complexes Ng@[3${}_{n}$]CP (3 ≤ *n* ≤ 5) based on cyclophanes (CP) having only one-carbon escape channels. It has been shown that in ten such cases, the Ng atom remained inside the cyclophane. However, in all these cases, without exception, the determined binding energy was positive, proving, as in the case of Ng@[3${}_{6}$]SP endohedral complexes, that the Ng⋯CP interaction inside the cage is always destabilizing. More importantly, in the remaining ten cases, the initially trapped Ng atom has spontaneously escaped from the inside of the cyclophane cage, forming a much more energetically stable exohedral complex [3${}_{n}$]CP⋯Ng. According to the author’s opinion, the spontaneous escape of the initially trapped Ng atom from the cyclophane cage is strong evidence that the Ng⋯C bond path is not stabilizing. The highly repulsive nature of the intra-cage interactions in the complexes with He has also been confirmed by *s*-isosurfaces obtained by the NCI (noncovalent interaction) method. Nevertheless, similar *s*-isosurfaces for the complexes with Ar or Kr have shown that the Ar/Kr⋯C interactions are weakly attractive. However, the NCI method does not refer to the energetics of the interaction itself.

In summary, the presented results, obtained on the basis of the study of the endohedral complexes Ng@[3${}_{6}$]SP and Ng@[3${}_{n}$]CP, therefore, have provided new strong arguments showing that Bader’s topological bond path does not necessarily indicate a stabilizing interaction. For this reason alone, contrary to what many still believe, it cannot also mean a chemical bond.

It is worth noting that, as expected, increasing the length of the side chains from two to three (i.e., from ethylene to trimethylene) has resulted in facilitating encapsulation and, as a consequence, obtaining more endohedral complexes (moreover, involving larger Ng atoms). It is, therefore, possible that even greater elongation of the aliphatic side chains would make it possible to obtain such endohedral complexes. Of course, difficulties in synthesizing a suitable cyclophane and/or its possible closure may be a problem. On the other hand, trapping can also be facilitated by inserting charged fragments.

## Figures and Tables

**Table 1:** The selected structural parameters (in Å or degrees) for the [2${}_{6}$] [52] and [3${}_{n}$]cyclophanes.

CP	Label	Symm.	$d_{\pi \cdots \pi }{\;}^{a}$	$d_{C_{r}C_{r}}$ ${}^{a}$	$d_{C_{r}C_{c}}$ ${}^{a}$	$d_{C_{c}C_{c}}$ ${}^{a}$	$\alpha_{C_{r}C_{c}C_{c}}$ ${}^{a}$	$\alpha_{C_{c}C_{c}C_{c}}$ ${}^{a}$
[2${}_{6}$](1,2,3,4,5,6)${\;}^{b}$	**1**	C${}_{1}$	2.654	1.406	1.520	1.592	110.3	n/a
[3${}_{6}$](1,2,3,4,5,6) ${}^{c}$		D${}_{6h}$	2.954	1.400	1.519	1.482	120.4	129.5
[3${}_{6}$](1,2,3,4,5,6) ${}^{d}$		D${}_{6h}$	2.919	1.403	1.515	1.567	120.4	124.7
[3${}_{6}$](1,2,3,4,5,6)	**2**	C${}_{6h}$	2.961	1.403	1.522	1.551	117.2	120.8
[3${}_{5}$](1,2,3,4,5)	**3a**	C${}_{s}$	2.958–3.204	1.385–1.409	1.517–1.521	1.544–1.549	114.0–117.5	119.0–120.9
[3${}_{5}$](1,2,3,4,5)	**3b**	C${}_{s}$	2.952–3.281	1.384–1.411	1.517–1.522	1.542–1.551	113.7–117.6	117.3–121.0
[3${}_{5}$](1,2,3,4,5)	**3c**	C${}_{s}$	2.950–3.279	1.385–1.411	1.517–1.524	1.542–1.550	113.9–117.2	118.8–121.2
[3${}_{5}$](1,2,3,4,5)	**3d**	C${}_{2v}$	2.942–3.136	1.387–1.408	1.518–1.519	1.544–1.573	117.2–121.1	118.7–124.0
[3${}_{4}$](1,2,3,5)	**4a**	C${}_{s}$	2.973–3.228	1.384–1.408	1.512–1.520	1.541–1.549	114.1–117.4	116.8–120.3
[3${}_{4}$](1,2,3,5)	**4b**	C${}_{s}$	2.940–3.156	1.385–1.407	1.513–1.518	1.543–1.570	114.2–121.5	117.5–123.5
[3${}_{4}$](1,2,4,5)	**5a**	D${}_{2h}$	3.075–3.247	1.392–1.400	1.517	1.541	114.2	116.9
[3${}_{4}$](1,2,4,5)	**5b**	D${}_{2}$	2.990–3.120	1.392–1.401	1.516–1.519	1.542–1.559	118.0–119.4	120.7
[3${}_{3}$](1,2,3)	**6a**	C${}_{3h}$	3.118–3.168	1.386–1.397	1.514	1.543	114.5	117.4
[3${}_{3}$](1,2,3)	**6b**	C${}_{s}$	3.104–3.212	1.390–1.393	1.514	1.542–1.544	114.4–114.5	117.1–117.6

${}^{a}$ Most of the considered systems feature diverse values, therefore, in these cases, they are given in the $v_{\min}$–$v_{\max}$ format. ${}^{b}$ Values from ref. [52]. ${}^{c}$ Experimental values taken from ref [72]. ${}^{d}$ The 7th-order saddle point, in line with [71].

**Table 2:** Binding and deformation energies (in kcal/mol) and selected structural parameters (in Å or degrees) for the [2${}_{6}$] [52] and [3${}_{6}$] superphanes and their endohedral complexes.

System	$E_{b}$	$E_{\mathrm{def}}$	$E_{\mathrm{def}}^{\%}$	$d_{\pi \cdots \pi }$ ${}^{a}$	$d_{C_{r}C_{r}}$ ${}^{a}$	$d_{C_{r}C_{c}}$ ${}^{a}$	$d_{C_{c}C_{c}}$ ${}^{a}$	$\alpha_{C_{r}C_{c}C_{c}}$ ${}^{a}$	$\alpha_{C_{c}C_{c}C_{c}}$ ${}^{a}$
[2${}_{6}$]SP	n/a	n/a	n/a	2.654	1.406	1.520	1.592	110.3	n/a
He@[2${}_{6}$]SP	80.7	8.5	10.5	2.819	1.417	1.528	1.601	113.3	n/a
Ne@[2${}_{6}$]SP	202.1	59.5	29.4	3.130	1.431	1.545	1.630	118.8	n/a
Ar@[2${}_{6}$]SP	438.4	187.6	42.8	3.568	1.447	1.575	1.707	126.1	n/a
Kr@[2${}_{6}$]SP	551.5	241.7	43.8	3.701–3.706	1.434–1.482	1.587	1.751–1.754	127.9	n/a
[3${}_{6}$]SP	n/a	n/a	n/a	2.961	1.403	1.522	1.551	117.2	120.8
He@[3${}_{6}$]SP	51.3	7.7	15.1	3.194	1.410	1.523	1.559	118.6	123.9
Ne@[3${}_{6}$]SP	120.7	43.7	36.2	3.560	1.416	1.528	1.574	120.8	128.8
Ar@[3${}_{6}$]SP	255.2	129.1	50.6	3.954–4.168	1.417–1.426	1.534–1.545	1.593–1.618	123.6–124.5	133.4–137.2
Kr@[3${}_{6}$]SP	315.7	166.9	52.9	4.228–4.249	1.424–1.425	1.544–1.545	1.618–1.622	125.2–125.3	137.5–137.9

${}^{a}$ Some of the considered systems feature diverse values, therefore, in these cases, they are given in the $v_{\min}$–$v_{\max}$ format.

**Table 3:** Binding and deformation energies (in kcal/mol) and selected structural parameters (in Å or degrees) for the obtained Ng@[3${}_{n}$]CP endohedral complexes ${}^{a}$.

Complex	$E_{b}$	$E_{\mathrm{def}}$	$E_{\mathrm{def}}^{\%}$	$d_{\pi \cdots \pi }$ ${}^{b}$	$d_{C_{r}C_{r}}$ ${}^{b}$	$d_{C_{r}C_{c}}$ ${}^{b}$	$d_{C_{c}C_{c}}$ ${}^{b}$	$\alpha_{C_{r}C_{c}C_{c}}$ ${}^{b}$	$\alpha_{C_{c}C_{c}C_{c}}$ ${}^{b}$
**3a**	n/a	n/a	n/a	2.958–3.204	1.385–1.409	1.517–1.521	1.544–1.549	114.0–117.5	119.0–120.9
He@**3a**	44.2	9.6	21.8	3.137–3.739	1.392–1.412	1.520–1.524	1.552–1.563	115.0–119.1	122.3–124.4
**4a**	n/a	n/a	n/a	2.973–3.228	1.384–1.408	1.512–1.520	1.541–1.549	114.1–117.4	116.8–120.3
He@**4a**	39.1	9.3	23.8	3.177–3.709	1.388–1.412	1.517–1.522	1.542–1.562	115.1–118.9	119.0–123.7
**5a**	n/a	n/a	n/a	3.075–3.247	1.392–1.400	1.517	1.541	114.2	116.9
He@**5a**	38.2	8.7	22.8	3.391–3.653	1.396–1.403	1.518	1.550	115.4	119.8
Ar@**5a**	170.1	86.0	50.5	4.237–4.698	1.407–1.410	1.536	1.591	119.4	127.5
Kr@**5a**	215.6	106.5	49.4	4.368–4.868	1.411–1.414	1.544	1.605	121.0	129.4
**6a**	n/a	n/a	n/a	3.118–3.168	1.386–1.397	1.514	1.543	114.5	117.4
He@**6a**	37.7	8.9	23.5	3.453–3.540	1.391–1.400	1.517	1.554	115.7–115.8	120.6
Ne@**6a**	79.8	36.3	45.5	3.839–4.039	1.394–1.404	1.524–1.527	1.569–1.575	116.7–116.8	123.6–124.5
Ar@**6a**	162.2	86.6	53.4	4.376–4.563	1.401–1.405	1.544–1.545	1.606–1.607	118.6–118.8	127.0–127.2
Kr@**6a**	204.2	107.1	52.5	4.526–4.722	1.406	1.555	1.624	120.7–120.8	129.1
**6b**	n/a	n/a	n/a	3.104–3.212	1.390–1.393	1.514	1.542–1.544	114.4–114.5	117.1–117.6
He@**6b**	37.5	8.9	23.8	3.406–3.642	1.394–1.397	1.516–1.518	1.551–1.557	115.6–115.7	119.7–121.2

${}^{a}$ The values of the structural parameters obtained for [3${}_{n}$]CPs were copied from Table 1 to simplify the visualization of the changes caused by the encapsulation. ${}^{b}$ Some of the considered systems feature diverse values, therefore, in these cases, they are given in the $v_{\min}$–$v_{\max}$ format.

## Data Availability

Data available from the author on reasonable request.

## Supplementary Materials

The following supporting information can be downloaded at: https://www.mdpi.com/article/10.3390/molecules28176353/s1, Cartesian coordinates of considered systems, Table S1: Values of ρ and $\nabla^{2}\rho$ (in a.u.) at BCPs of Ng⋯C${}_{r}$, Ng⋯π, and Ng⋯C${}_{c}$ interactions.

## Abbreviations

The following abbreviations are used in this manuscript:

QTAIM	Quantum theory of atoms in molecules
BP	Bond path
BCP	Bond critical point
Ng	Noble gas atom
SP	Superphane
CP	Cyclophane
NCI	Noncovalent interaction index
