# Supplementary material for: Bader’s Topological Bond Path Does Not Necessarily Indicate Stabilizing Interaction—Proof Studies Based on the Ng@[3n]cyclophane Endohedral Complexes
Source: Molecules. 2023 Aug 30;28(17):6353. doi: 10.3390/molecules28176353 (PMC10490063; doi:10.3390/molecules28176353)
Supplement: Supplementary file 1 [file molecules-28-06353-s001.zip › molecules-2555014-supplementary.pdf]

Bader's Topological Bond Path Does Not Necessarily Indicate Stabilizing Interaction–Proof Studies Based on the Ng@[3_n]cyclophane Endohedral Complexes

Mirosław Jabłoński

Faculty of Chemistry, Nicolaus Copernicus University in Toruń, Gagarina 7, 87-100 Toruń, Poland
teojab@chem.umk.pl; Tel.: +48-056-611-4695

SUPPORTING INFORMATION

Cartesian coordinates of considered systems

I) Cyclophanes

[3_6](1,2,3,4,5,6) – D_{6h}

6	0.000000	1.402998	1.459253
6	-1.215032	0.701499	1.459253
6	1.215032	0.701499	1.459253
6	0.000000	1.402998	-1.459253
6	1.215032	0.701499	-1.459253
6	-1.215032	0.701499	-1.459253
6	-0.000000	-1.402998	-1.459253
6	1.215032	-0.701499	-1.459253
6	-1.215032	-0.701499	-1.459253
6	-0.000000	-1.402998	1.459253
6	-1.215032	-0.701499	1.459253
6	1.215032	-0.701499	1.459253
6	0.000000	3.643602	-0.000000
1	0.866164	4.309989	0.000000
1	-0.866164	4.309989	-0.000000
6	0.000000	2.915869	1.388077
1	-0.857592	3.290466	1.944593
1	0.857592	3.290466	1.944593
6	0.000000	2.915869	-1.388077
1	0.857592	3.290466	-1.944593
1	-0.857592	3.290466	-1.944593
6	2.525216	1.457934	1.388077
1	3.278423	0.902536	1.944593
1	2.420831	2.387929	1.944593
6	3.155452	1.821801	-0.000000
1	4.165642	1.404875	0.000000
1	3.299478	2.905114	-0.000000
6	2.525216	1.457934	-1.388077
1	3.278423	0.902536	-1.944593
1	2.420831	2.387929	-1.944593
6	2.525216	-1.457934	1.388077
1	3.278423	-0.902536	1.944593
1	2.420831	-2.387929	1.944593
6	3.155452	-1.821801	-0.000000
1	3.299478	-2.905114	0.000000
1	4.165642	-1.404875	-0.000000
6	2.525216	-1.457934	-1.388077
1	2.420831	-2.387929	-1.944593
1	3.278423	-0.902536	-1.944593
6	-0.000000	-2.915869	1.388077
1	-0.857592	-3.290466	1.944593
1	0.857592	-3.290466	1.944593
6	-0.000000	-3.643602	-0.000000
1	-0.866164	-4.309989	0.000000
1	0.866164	-4.309989	-0.000000
6	-0.000000	-2.915869	-1.388077
1	-0.857592	-3.290466	-1.944593
1	0.857592	-3.290466	-1.944593
6	-2.525216	-1.457934	1.388077
1	-3.278423	-0.902536	1.944593
1	-2.420831	-2.387929	1.944593
6	-3.155452	-1.821801	-0.000000
1	-4.165642	-1.404875	0.000000

1	-3.299478	-2.905114	-0.000000
6	-2.525216	-1.457934	-1.388077
1	-3.278423	-0.902536	-1.944593
1	-2.420831	-2.387929	-1.944593
6	-2.525216	1.457934	1.388077
1	-2.420831	2.387929	1.944593
1	-3.278423	0.902536	1.944593
6	-3.155452	1.821801	-0.000000
1	-3.299478	2.905114	0.000000
1	-4.165642	1.404875	-0.000000
6	-2.525216	1.457934	-1.388077
1	-2.420831	2.387929	-1.944593
1	-3.278423	0.902536	-1.944593

$[3_6](1,2,3,4,5,6) – C_{6h}$ (**2**)

6	0.000000	1.403170	1.480722
6	-1.215181	0.701585	1.480722
6	1.215181	0.701585	1.480722
6	0.000000	1.403170	-1.480722
6	1.215181	0.701585	-1.480722
6	-1.215181	0.701585	-1.480722
6	-0.000000	-1.403170	-1.480722
6	1.215181	-0.701585	-1.480722
6	-1.215181	-0.701585	-1.480722
6	-0.000000	-1.403170	1.480722
6	-1.215181	-0.701585	1.480722
6	1.215181	-0.701585	1.480722
6	0.470768	3.516724	0.000000
1	1.560421	3.514151	0.000000
1	0.188585	4.575486	0.000000
6	-0.007808	2.919131	1.348643
1	-1.001244	3.298780	1.575116
1	0.630780	3.352704	2.125735
6	-0.007808	2.919131	-1.348643
1	0.630780	3.352704	-2.125735
1	-1.001244	3.298780	-1.575116
6	2.524138	1.466328	1.348643
1	3.218917	1.130081	2.125735
1	2.356205	2.516493	1.575116
6	3.280956	1.350664	0.000000
1	3.823554	0.405712	0.000000
1	4.056779	2.124423	0.000000
6	2.524138	1.466328	-1.348643
1	3.218917	1.130081	-2.125735
1	2.356205	2.516493	-1.575116
6	2.531946	-1.452804	1.348643
1	3.357449	-0.782287	1.575116
1	2.588137	-2.222624	2.125735
6	2.810188	-2.166059	0.000000
1	2.263134	-3.108439	0.000000
1	3.868194	-2.451062	0.000000
6	2.531946	-1.452804	-1.348643
1	2.588137	-2.222624	-2.125735
1	3.357449	-0.782287	-1.575116
6	0.007808	-2.919131	1.348643
1	-0.630780	-3.352704	2.125735
1	1.001244	-3.298780	1.575116
6	-0.470768	-3.516724	0.000000
1	-1.560421	-3.514151	0.000000
1	-0.188585	-4.575486	0.000000

6	0.007808	-2.919131	-1.348643
1	-0.630780	-3.352704	-2.125735
1	1.001244	-3.298780	-1.575116
6	-2.524138	-1.466328	1.348643
1	-3.218917	-1.130081	2.125735
1	-2.356205	-2.516493	1.575116
6	-3.280956	-1.350664	0.000000
1	-3.823554	-0.405712	0.000000
1	-4.056779	-2.124423	0.000000
6	-2.524138	-1.466328	-1.348643
1	-3.218917	-1.130081	-2.125735
1	-2.356205	-2.516493	-1.575116
6	-2.531946	1.452804	1.348643
1	-2.588137	2.222624	2.125735
1	-3.357449	0.782287	1.575116
6	-2.810188	2.166059	0.000000
1	-2.263134	3.108439	0.000000
1	-3.868194	2.451062	0.000000
6	-2.531946	1.452804	-1.348643
1	-2.588137	2.222624	-2.125735
1	-3.357449	0.782287	-1.575116

$[3_5](1,2,3,4,5)$ – C_s (**3a**)

6	1.696283	-0.076078	1.601782
6	1.100937	1.171955	1.516432
6	0.976539	-1.260422	1.533217
6	1.696283	-0.076078	-1.601782
6	0.976539	-1.260422	-1.533217
6	1.100937	1.171955	-1.516432
6	-1.054420	0.058369	-1.481635
6	-0.419334	-1.199536	-1.485873
6	-0.299653	1.245062	-1.478754
6	-1.054420	0.058369	1.481635
6	-0.299653	1.245062	1.478754
6	-0.419334	-1.199536	1.485873
6	1.775687	-2.535464	1.339874
1	1.153042	-3.422658	1.456989
1	2.528381	-2.596999	2.134068
6	2.548403	-2.596288	0.000000
1	3.136642	-3.520820	0.000000
1	3.277277	-1.778785	0.000000
6	1.775687	-2.535464	-1.339874
1	1.153042	-3.422658	-1.456989
1	2.528381	-2.596999	-2.134068
6	-1.225944	-2.479493	1.347263
1	-0.899477	-3.183485	2.120865
1	-2.271771	-2.283651	1.576542
6	-1.124230	-3.236536	0.000000
1	-1.906300	-4.004055	0.000000
1	-0.185190	-3.788970	0.000000
6	-1.225944	-2.479493	-1.347263
1	-2.271771	-2.283651	-1.576542
1	-0.899477	-3.183485	-2.120865
6	-2.567310	0.139017	1.346097
1	-2.897806	1.151989	1.565836
1	-3.030683	-0.474992	2.126053
6	-3.182617	-0.319404	0.000000
1	-4.230996	-0.001011	0.000000
1	-3.216531	-1.408398	0.000000
6	-2.567310	0.139017	-1.346097

1	-2.897806	1.151989	-1.565836
1	-3.030683	-0.474992	-2.126053
6	-0.967916	2.603623	1.343532
1	-0.235995	3.378439	1.567272
1	-1.726541	2.711854	2.126573
6	-1.667367	2.926136	0.000000
1	-1.891547	3.998627	0.000000
1	-2.639493	2.432985	0.000000
6	-0.967916	2.603623	-1.343532
1	-0.235995	3.378439	-1.567272
1	-1.726541	2.711854	-2.126573
6	2.018281	2.368932	1.330774
1	3.047266	2.011922	1.439089
1	1.871110	3.094186	2.140144
6	1.910803	3.145263	0.000000
1	2.718201	3.886221	0.000000
1	0.990861	3.728339	0.000000
6	2.018281	2.368932	-1.330774
1	3.047266	2.011922	-1.439089
1	1.871110	3.094186	-2.140144
1	2.782140	-0.131975	1.608698
1	2.782140	-0.131975	-1.608698

$[3_5](1,2,3,4,5) - C_s$ (**3b**)

6	-1.653192	0.005645	1.640453
6	-1.037116	-1.234223	1.535564
6	-0.960941	1.200724	1.545858
6	-1.653192	0.005645	-1.640453
6	-0.960941	1.200724	-1.545858
6	-1.037116	-1.234223	-1.535564
6	1.094266	-0.078710	-1.482005
6	0.437744	1.164958	-1.485933
6	0.362477	-1.284974	-1.475975
6	1.094266	-0.078710	1.482005
6	0.362477	-1.284974	1.475975
6	0.437744	1.164958	1.485933
6	-1.786883	2.457010	1.342863
1	-1.186074	3.359598	1.456605
1	-2.544088	2.505903	2.133636
6	-2.558004	2.491069	0.000000
1	-3.177179	3.395221	0.000000
1	-3.258116	1.648478	0.000000
6	-1.786883	2.457010	-1.342863
1	-1.186074	3.359598	-1.456605
1	-2.544088	2.505903	-2.133636
6	1.217597	2.461116	1.345351
1	0.879898	3.157186	2.121422
1	2.268350	2.287197	1.569297
6	1.095439	3.217037	0.000000
1	1.858125	4.003812	0.000000
1	0.142848	3.745659	0.000000
6	1.217597	2.461116	-1.345351
1	2.268350	2.287197	-1.569297
1	0.879898	3.157186	-2.121422
6	2.609051	-0.126293	1.350102
1	2.962692	-1.127069	1.587832
1	3.053890	0.509794	2.122914
6	3.219094	0.332801	0.000000
1	4.270763	0.025308	0.000000
1	3.242111	1.422337	0.000000

6	2.609051	-0.126293	-1.350102
1	2.962692	-1.127069	-1.587832
1	3.053890	0.509794	-2.122914
6	1.070879	-2.622927	1.323245
1	0.354535	-3.427555	1.477585
1	1.796882	-2.740474	2.135830
6	1.815857	-2.889928	0.000000
1	2.109256	-3.945733	0.000000
1	2.752242	-2.334709	0.000000
6	1.070879	-2.622927	-1.323245
1	0.354535	-3.427555	-1.477585
1	1.796882	-2.740474	-2.135830
6	-1.966817	-2.416779	1.318046
1	-1.442083	-3.371374	1.353444
1	-2.692282	-2.445354	2.138765
6	-2.765955	-2.344615	0.000000
6	-1.966817	-2.416779	-1.318046
1	-2.692282	-2.445354	-2.138765
1	-1.442083	-3.371374	-1.353444
1	-2.739886	0.041305	1.677124
1	-2.739886	0.041305	-1.677124
1	-3.365806	-1.428672	0.000000
1	-3.481280	-3.174771	0.000000

$[3_5](1,2,3,4,5) – C_s$ (**3c**)

6	1.603401	0.177007	1.639472
6	0.832769	1.323259	1.544008
6	1.064795	-1.096280	1.543771
6	1.603401	0.177007	-1.639472
6	1.064795	-1.096280	-1.543771
6	0.832769	1.323259	-1.544008
6	-1.141767	-0.083999	-1.474835
6	-0.325524	-1.234990	-1.484586
6	-0.563547	1.202914	-1.478243
6	-1.141767	-0.083999	1.474835
6	-0.563547	1.202914	1.478243
6	-0.325524	-1.234990	1.484586
6	2.042582	-2.238374	1.341162
1	1.560276	-3.209924	1.450178
1	2.797853	-2.193215	2.134046
6	2.813783	-2.172382	0.000000
1	3.543031	-2.990312	0.000000
1	3.400987	-1.247553	0.000000
6	2.042582	-2.238374	-1.341162
1	1.560276	-3.209924	-1.450178
1	2.797853	-2.193215	-2.134046
6	-0.927711	-2.623835	1.350448
1	-0.484899	-3.268631	2.117594
1	-1.987082	-2.594369	1.596685
6	-0.724669	-3.357175	0.000000
1	-1.391104	-4.226992	0.000000
1	0.282698	-3.772575	0.000000
6	-0.927711	-2.623835	-1.350448
1	-1.987082	-2.594369	-1.596685
1	-0.484899	-3.268631	-2.117594
6	-2.648822	-0.254854	1.328421
1	-3.152593	0.693569	1.492515
1	-3.014133	-0.901839	2.133662
6	-3.166837	-0.845871	0.000000
1	-4.256781	-0.732767	0.000000

1	-2.993406	-1.919971	0.000000
6	-2.648822	-0.254854	-1.328421
1	-3.152593	0.693569	-1.492515
1	-3.014133	-0.901839	-2.133662
6	-1.396437	2.468100	1.328804
1	-1.118041	3.163245	2.128975
1	-2.449279	2.259740	1.500417
6	-1.268069	3.240415	0.000000
1	-2.041137	4.017133	0.000000
1	-0.323631	3.780427	0.000000
6	-1.396437	2.468100	-1.328804
1	-1.118041	3.163245	-2.128975
1	-2.449279	2.259740	-1.500417
6	1.586046	2.626281	1.347208
1	0.940159	3.493974	1.479393
1	2.346944	2.705179	2.131939
6	2.347675	2.714197	0.000000
6	1.586046	2.626281	-1.347208
1	2.346944	2.705179	-2.131939
1	0.940159	3.493974	-1.479393
1	2.685849	0.281484	1.676061
1	2.685849	0.281484	-1.676061
1	3.101840	1.919563	0.000000
1	2.906690	3.656777	0.000000

[3_5](1,2,3,4,5) – C_{2V} (**3d**)

6	1.567789	-0.000000	-1.779525
6	1.505435	1.219015	-1.121380
6	1.505435	-1.219015	-1.121380
6	-1.567789	0.000000	-1.779525
6	-1.505435	-1.219015	-1.121380
6	-1.505435	1.219015	-1.121380
6	-1.471106	0.000000	0.974885
6	-1.473996	-1.223827	0.279202
6	-1.473996	1.223827	0.279202
6	1.471106	-0.000000	0.974885
6	1.473996	1.223827	0.279202
6	1.473996	-1.223827	0.279202
6	1.328057	-2.461411	-1.977471
1	2.141225	-3.173603	-1.792869
1	1.435584	-2.156820	-3.023240
6	0.000000	-3.234839	-1.830102
1	0.000000	-3.767599	-0.880005
1	0.000000	-4.017277	-2.597288
6	-1.328057	-2.461411	-1.977471
1	-2.141225	-3.173603	-1.792869
1	-1.435584	-2.156820	-3.023240
6	1.346605	-2.545745	1.014106
1	1.583630	-3.354169	0.323729
1	2.121477	-2.606842	1.786349
6	0.000000	-2.844089	1.720719
1	0.000000	-2.323286	2.674165
1	0.000000	-3.908529	1.980092
6	-1.346605	-2.545745	1.014106
1	-2.121477	-2.606842	1.786349
1	-1.583630	-3.354169	0.323729
6	1.388758	-0.000000	2.490981
1	1.945232	0.864236	2.858083
1	1.945232	-0.864236	2.858083
6	0.000000	0.000000	3.228869

1	0.000000	0.857970	3.903197
1	-0.000000	-0.857970	3.903197
6	-1.388758	0.000000	2.490981
1	-1.945232	0.864236	2.858083
1	-1.945232	-0.864236	2.858083
6	1.346605	2.545745	1.014106
1	1.583630	3.354169	0.323729
1	2.121477	2.606842	1.786349
6	0.000000	2.844089	1.720719
1	0.000000	3.908529	1.980092
1	0.000000	2.323286	2.674165
6	-1.346605	2.545745	1.014106
1	-1.583630	3.354169	0.323729
1	-2.121477	2.606842	1.786349
6	1.328057	2.461411	-1.977471
1	1.435584	2.156820	-3.023240
1	2.141225	3.173603	-1.792869
6	0.000000	3.234839	-1.830102
1	0.000000	4.017277	-2.597288
1	0.000000	3.767599	-0.880005
6	-1.328057	2.461411	-1.977471
1	-1.435584	2.156820	-3.023240
1	-2.141225	3.173603	-1.792869
1	1.552099	-0.000000	-2.866106
1	-1.552099	0.000000	-2.866106

$[3_4](1,2,3,5) – C_s$ (**4a**)

6	1.096853	1.050193	1.613828
6	-0.108647	1.738784	1.570228
6	1.156888	-0.335209	1.537775
6	1.096853	1.050193	-1.613828
6	1.156888	-0.335209	-1.537775
6	-0.108647	1.738784	-1.570228
6	-1.258652	-0.407553	-1.522196
6	-0.031430	-1.089397	-1.486603
6	-1.269347	0.985978	-1.590416
6	-1.258652	-0.407553	1.522196
6	-1.269347	0.985978	1.590416
6	-0.031430	-1.089397	1.486603
6	2.536058	-0.939756	1.343087
1	2.522374	-2.021696	1.473317
1	3.193087	-0.558056	2.132588
6	3.208698	-0.563567	0.000000
6	2.536058	-0.939756	-1.343087
1	2.522374	-2.021696	-1.473317
1	3.193087	-0.558056	-2.132588
6	0.029187	-2.599087	1.325576
1	0.645635	-3.013522	2.131709
1	-0.960036	-3.025466	1.484364
6	0.590701	-3.149360	0.000000
6	0.029187	-2.599087	-1.325576
1	-0.960036	-3.025466	-1.484364
1	0.645635	-3.013522	-2.131709
6	-2.612067	-1.067498	1.312539
1	-3.270844	-0.780568	2.139676
1	-2.546746	-2.154889	1.348614
6	-3.311718	-0.663809	0.000000
6	-2.612067	-1.067498	-1.312539
1	-3.270844	-0.780568	-2.139676
1	-2.546746	-2.154889	-1.348614
6	-0.141457	3.230666	1.323702

1	0.371095	3.755287	2.138242
1	-1.180913	3.576465	1.337222
6	0.523747	3.669772	0.000000
6	-0.141457	3.230666	-1.323702
1	0.371095	3.755287	-2.138242
1	-1.180913	3.576465	-1.337222
1	2.027993	1.612047	1.598409
1	2.027993	1.612047	-1.598409
1	-2.225611	1.501390	1.549691
1	-2.225611	1.501390	-1.549691
1	0.562720	4.764607	0.000000
1	1.566312	3.333109	0.000000
1	3.358654	0.521472	0.000000
1	4.212724	-1.002504	0.000000
1	1.672537	-3.030068	0.000000
1	0.422283	-4.232172	0.000000
1	-3.475900	0.418285	0.000000
1	-4.308608	-1.118387	0.000000

$[3_4](1,2,3,5) – C_s$ (**4b**)

6	1.320771	1.022810	1.558309
6	0.211416	1.851948	1.549464
6	1.212812	-0.365092	1.500198
6	1.320771	1.022810	-1.558309
6	1.212812	-0.365092	-1.500198
6	0.211416	1.851948	-1.549464
6	-1.195259	-0.132113	-1.505863
6	-0.058324	-0.960240	-1.470147
6	-1.038154	1.247577	-1.578182
6	-1.195259	-0.132113	1.505863
6	-1.038154	1.247577	1.578182
6	-0.058324	-0.960240	1.470147
6	2.501095	-1.148656	1.326335
1	2.613257	-1.877127	2.138613
1	3.332610	-0.446278	1.440754
6	2.683449	-1.915063	0.000000
1	2.035563	-2.786949	0.000000
1	3.702798	-2.316831	0.000000
6	2.501095	-1.148656	-1.326335
1	2.613257	-1.877127	-2.138613
1	3.332610	-0.446278	-1.440754
6	-0.203726	-2.467551	1.383682
1	0.624767	-2.909261	1.942885
1	-1.100342	-2.743579	1.944122
6	-0.275598	-3.206800	0.000000
1	-1.195241	-3.795110	0.000000
1	0.513874	-3.960820	0.000000
6	-0.203726	-2.467551	-1.383682
1	-1.100342	-2.743579	-1.944122
1	0.624767	-2.909261	-1.942885
6	-2.608525	-0.656961	1.328691
1	-3.291767	0.190256	1.443737
1	-2.859022	-1.353256	2.138331
6	-2.932026	-1.372382	0.000000
1	-4.009375	-1.571965	0.000000
1	-2.463931	-2.352469	0.000000
6	-2.608525	-0.656961	-1.328691
1	-3.291767	0.190256	-1.443737
1	-2.859022	-1.353256	-2.138331
6	0.346877	3.340869	1.319893

1	1.407749	3.613543	1.339166
1	-0.129738	3.889773	2.140160
6	-0.281507	3.837617	0.000000
1	-0.229543	4.931935	0.000000
1	-1.348130	3.588386	0.000000
6	0.346877	3.340869	-1.319893
1	1.407749	3.613543	-1.339166
1	-0.129738	3.889773	-2.140160
1	2.309832	1.470089	1.504283
1	2.309832	1.470089	-1.504283
1	-1.927217	1.872098	1.546840
1	-1.927217	1.872098	-1.546840

[3_4](1,2,4,5) – D_{2h} (**5a**)

6	0.000000	1.351382	1.623608
6	-1.227030	0.699972	1.537596
6	1.227030	0.699972	1.537596
6	0.000000	1.351382	-1.623608
6	1.227030	0.699972	-1.537596
6	-1.227030	0.699972	-1.537596
6	-0.000000	-1.351382	-1.623608
6	1.227030	-0.699972	-1.537596
6	-1.227030	-0.699972	-1.537596
6	-0.000000	-1.351382	1.623608
6	-1.227030	-0.699972	1.537596
6	1.227030	-0.699972	1.537596
6	2.458296	1.556442	1.313187
1	3.369737	0.956746	1.343473
1	2.543671	2.271615	2.139088
6	2.436879	2.362806	0.000000
6	2.458296	1.556442	-1.313187
1	3.369737	0.956746	-1.343473
1	2.543671	2.271615	-2.139088
6	2.458296	-1.556442	1.313187
1	3.369737	-0.956746	1.343473
1	2.543671	-2.271615	2.139088
6	2.436879	-2.362806	-0.000000
6	2.458296	-1.556442	-1.313187
1	2.543671	-2.271615	-2.139088
1	3.369737	-0.956746	-1.343473
6	-2.458296	-1.556442	1.313187
1	-3.369737	-0.956746	1.343473
1	-2.543671	-2.271615	2.139088
6	-2.436879	-2.362806	0.000000
6	-2.458296	-1.556442	-1.313187
1	-3.369737	-0.956746	-1.343473
1	-2.543671	-2.271615	-2.139088
6	-2.458296	1.556442	1.313187
1	-2.543671	2.271615	2.139088
1	-3.369737	0.956746	1.343473
6	-2.436879	2.362806	-0.000000
6	-2.458296	1.556442	-1.313187
1	-2.543671	2.271615	-2.139088
1	-3.369737	0.956746	-1.343473
1	0.000000	2.439302	1.627830
1	0.000000	2.439302	-1.627830
1	-0.000000	-2.439302	1.627830
1	-0.000000	-2.439302	-1.627830
1	-3.309694	-3.025213	0.000000
1	-1.559945	-3.018810	0.000000

1	-3.309694	3.025213	0.000000
1	-1.559945	3.018810	0.000000
1	1.559945	3.018810	0.000000
1	3.309694	3.025213	0.000000
1	1.559945	-3.018810	0.000000
1	3.309694	-3.025213	0.000000

$[3_4](1,2,4,5) – D_2$ (**5b**)

6	-0.148075	1.337699	1.552915
6	-1.302487	0.562268	1.500850
6	1.145805	0.829268	1.472748
6	0.148075	1.337699	-1.552915
6	1.302487	0.562268	-1.500850
6	-1.145805	0.829268	-1.472748
6	-0.148075	-1.337699	-1.552915
6	1.145805	-0.829268	-1.472748
6	-1.302487	-0.562268	-1.500850
6	0.148075	-1.337699	1.552915
6	-1.145805	-0.829268	1.472748
6	1.302487	-0.562268	1.500850
6	2.264665	1.826681	1.243235
1	2.906013	1.880401	2.132033
1	1.803055	2.814445	1.150750
6	3.202665	1.597654	0.040882
1	3.908175	0.818162	0.316355
1	3.815446	2.500200	-0.054450
6	2.622782	1.302484	-1.375926
1	3.399692	0.777233	-1.941854
1	2.487258	2.261230	-1.885141
6	2.622782	-1.302484	1.375926
1	3.399692	-0.777233	1.941854
1	2.487258	-2.261230	1.885141
6	3.202665	-1.597654	-0.040882
1	3.815446	-2.500200	0.054450
1	3.908175	-0.818162	-0.316355
6	2.264665	-1.826681	-1.243235
1	1.803055	-2.814445	-1.150750
1	2.906013	-1.880401	-2.132033
6	-2.264665	-1.826681	1.243235
1	-2.906013	-1.880401	2.132033
1	-1.803055	-2.814445	1.150750
6	-3.202665	-1.597654	0.040882
1	-3.908175	-0.818162	0.316355
1	-3.815446	-2.500200	-0.054450
6	-2.622782	-1.302484	-1.375926
1	-3.399692	-0.777233	-1.941854
1	-2.487258	-2.261230	-1.885141
6	-2.622782	1.302484	1.375926
1	-2.487258	2.261230	1.885141
1	-3.399692	0.777233	1.941854
6	-3.202665	1.597654	-0.040882
1	-3.815446	2.500200	0.054450
1	-3.908175	0.818162	-0.316355
6	-2.264665	1.826681	-1.243235
1	-1.803055	2.814445	-1.150750
1	-2.906013	1.880401	-2.132033
1	-0.269248	2.418324	1.532468
1	0.269248	2.418324	-1.532468
1	0.269248	-2.418324	1.532468
1	-0.269248	-2.418324	-1.532468

$[3_3](1,2,3) – C_{3h}$ (**6a**)

6	1.298587	0.468974	1.584210
6	0.240962	1.380668	1.558949
6	1.075213	-0.899013	1.558949
6	1.298587	0.468974	-1.584210
6	1.075213	-0.899013	-1.558949
6	0.240962	1.380668	-1.558949
6	-1.316175	-0.481655	-1.558949
6	-0.243150	-1.359096	-1.584210
6	-1.055436	0.890122	-1.584210
6	-1.316175	-0.481655	1.558949
6	-1.055436	0.890122	1.584210
6	-0.243150	-1.359096	1.584210
6	2.214496	-1.866280	1.318427
1	2.263750	-2.590737	2.139194
1	3.162180	-1.316962	1.332609
6	2.110396	-2.661774	0.000000
1	1.175344	-3.231851	0.000000
1	2.913310	-3.406676	0.000000
6	2.214496	-1.866280	-1.318427
1	2.263750	-2.590737	-2.139194
1	3.162180	-1.316962	-1.332609
6	-2.723494	-0.984670	1.318427
1	-3.375519	-0.665097	2.139194
1	-2.721612	-2.080048	1.332609
6	-3.360362	-0.496769	0.000000
1	-3.386537	0.598047	0.000000
1	-4.406923	-0.819662	0.000000
6	-2.723494	-0.984670	-1.318427
1	-3.375519	-0.665097	-2.139194
1	-2.721612	-2.080048	-1.332609
6	0.508998	2.850950	1.318427
1	1.111769	3.255834	2.139194
1	-0.440568	3.397009	1.332609
6	1.249966	3.158543	0.000000
1	2.211193	2.633803	0.000000
1	1.493613	4.226338	0.000000
6	0.508998	2.850950	-1.318427
1	1.111769	3.255834	-2.139194
1	-0.440568	3.397009	-1.332609
1	2.318833	0.836782	1.511663
1	2.318833	0.836782	-1.511663
1	-1.884091	1.589778	1.511663
1	-1.884091	1.589778	-1.511663
1	-0.434742	-2.426559	1.511663
1	-0.434742	-2.426559	-1.511663

$[3_3](1,2,3) – C_s$ (**6b**)

6	0.307896	-1.280126	1.605841
6	1.301569	-0.304510	1.566354
6	-1.039731	-0.942275	1.560444
6	0.307896	-1.280126	-1.605841
6	-1.039731	-0.942275	-1.560444
6	1.301569	-0.304510	-1.566354
6	-0.420923	1.405804	-1.552141
6	-1.386524	0.406460	-1.565162
6	0.920728	1.031790	-1.583794
6	-0.420923	1.405804	1.552141

6	0.920728	1.031790	1.583794
6	-1.386524	0.406460	1.565162
6	-2.088288	-2.006918	1.318408
1	-3.081478	-1.545019	1.331700
1	-2.073726	-2.732960	2.139159
6	-1.911085	-2.789293	0.000000
1	-2.641145	-3.605964	0.000000
1	-0.926034	-3.268393	0.000000
6	-2.088288	-2.006918	-1.318408
1	-3.081478	-1.545019	-1.331700
1	-2.073726	-2.732960	-2.139159
6	-0.807172	2.850109	1.315698
1	-0.436832	3.470394	2.139790
1	-1.899041	2.937332	1.329408
6	-0.268807	3.448728	0.000000
1	0.824640	3.386825	0.000000
1	-0.507604	4.517717	0.000000
6	-0.807172	2.850109	-1.315698
1	-0.436832	3.470394	-2.139790
1	-1.899041	2.937332	-1.329408
6	2.743819	-0.693699	1.320740
1	3.100088	-1.329984	2.138838
1	3.367887	0.206467	1.334277
6	2.983628	-1.456788	0.000000
1	2.375252	-2.367592	0.000000
1	4.025822	-1.794026	0.000000
6	2.743819	-0.693699	-1.320740
1	3.100088	-1.329984	-2.138838
1	3.367887	0.206467	-1.334277
1	0.593263	-2.327965	1.555598
1	0.593263	-2.327965	-1.555598
1	1.688617	1.797542	1.510271
1	1.688617	1.797542	-1.510271
1	-2.433698	0.682162	1.471617
1	-2.433698	0.682162	-1.471617

II) Ng@cyclophane endohedral complexes

He@[3_6](1,2,3,4,5,6)

6	0.000000	-1.409536	1.596801
6	1.220694	-0.704768	1.596801
6	-1.220694	-0.704768	1.596801
6	0.000000	-1.409536	-1.596801
6	-1.220694	-0.704768	-1.596801
6	1.220694	-0.704768	-1.596801
6	0.000000	1.409536	-1.596801
6	-1.220694	0.704768	-1.596801
6	1.220694	0.704768	-1.596801
6	0.000000	1.409536	1.596801
6	1.220694	0.704768	1.596801
6	-1.220694	0.704768	1.596801
6	-0.468319	-3.472247	0.000000
1	-1.556696	-3.448924	0.000000
1	-0.211239	-4.537955	0.000000
6	0.009690	-2.916641	1.375520
1	1.000643	-3.307656	1.593858
1	-0.635612	-3.392174	2.122372
6	0.009690	-2.916641	-1.375520
1	-0.635612	-3.392174	-2.122372

1	1.000643	-3.307656	-1.593858
6	-2.521040	-1.466712	1.375520
1	-3.255514	-1.145631	2.122372
1	-2.364193	-2.520410	1.593858
6	-3.241213	-1.330547	0.000000
1	-3.765204	-0.376324	0.000000
1	-4.035604	-2.086039	0.000000
6	-2.521040	-1.466712	-1.375520
1	-3.255514	-1.145631	-2.122372
1	-2.364193	-2.520410	-1.593858
6	-2.530730	1.449928	1.375520
1	-3.364835	0.787246	1.593858
1	-2.619903	2.246543	2.122372
6	-2.772894	2.141700	0.000000
1	-2.208508	3.072601	0.000000
1	-3.824365	2.451916	0.000000
6	-2.530730	1.449928	-1.375520
1	-2.619903	2.246543	-2.122372
1	-3.364835	0.787246	-1.593858
6	-0.009690	2.916641	1.375520
1	0.635612	3.392174	2.122372
1	-1.000643	3.307656	1.593858
6	0.468319	3.472247	0.000000
1	1.556696	3.448924	0.000000
1	0.211239	4.537955	0.000000
6	-0.009690	2.916641	-1.375520
1	0.635612	3.392174	-2.122372
1	-1.000643	3.307656	-1.593858
6	2.521040	1.466712	1.375520
1	3.255514	1.145631	2.122372
1	2.364193	2.520410	1.593858
6	3.241213	1.330547	0.000000
1	3.765204	0.376324	0.000000
1	4.035604	2.086039	0.000000
6	2.521040	1.466712	-1.375520
1	3.255514	1.145631	-2.122372
1	2.364193	2.520410	-1.593858
6	2.530730	-1.449928	1.375520
1	2.619903	-2.246543	2.122372
1	3.364835	-0.787246	1.593858
6	2.772894	-2.141700	0.000000
1	2.208508	-3.072601	0.000000
1	3.824365	-2.451916	0.000000
6	2.530730	-1.449928	-1.375520
1	2.619903	-2.246543	-2.122372
1	3.364835	-0.787246	-1.593858
2	0.000000	0.000000	0.000000

Ne@[3_6](1,2,3,4,5,6)

6	-0.000000	-1.416009	1.780171
6	1.226300	-0.708005	1.780171
6	-1.226300	-0.708005	1.780171
6	-0.000000	-1.416009	-1.780171
6	-1.226300	-0.708005	-1.780171
6	1.226300	-0.708005	-1.780171
6	0.000000	1.416009	-1.780171
6	-1.226300	0.708005	-1.780171
6	1.226300	0.708005	-1.780171
6	0.000000	1.416009	1.780171
6	1.226300	0.708005	1.780171

6	-1.226300	0.708005	1.780171
6	-0.464328	-3.388092	0.000000
1	-1.549345	-3.332084	0.000000
1	-0.245505	-4.462771	0.000000
6	0.010698	-2.900617	1.419327
1	0.995325	-3.313252	1.626406
1	-0.649377	-3.434864	2.111838
6	0.010698	-2.900617	-1.419327
1	-0.649377	-3.434864	-2.111838
1	0.995325	-3.313252	-1.626406
6	-2.506660	-1.459573	1.419327
1	-3.299368	-1.155055	2.111838
1	-2.371698	-2.518603	1.626406
6	-3.166338	-1.291927	0.000000
1	-3.660342	-0.324270	0.000000
1	-3.987626	-2.018772	0.000000
6	-2.506660	-1.459573	-1.419327
1	-3.299368	-1.155055	-2.111838
1	-2.371698	-2.518603	-1.626406
6	-2.517357	1.441044	1.419327
1	-3.367023	0.794649	1.626406
1	-2.649991	2.279809	2.111838
6	-2.702010	2.096165	0.000000
1	-2.110997	3.007814	0.000000
1	-3.742121	2.443999	0.000000
6	-2.517357	1.441044	-1.419327
1	-2.649991	2.279809	-2.111838
1	-3.367023	0.794649	-1.626406
6	-0.010698	2.900617	1.419327
1	0.649377	3.434864	2.111838
1	-0.995325	3.313252	1.626406
6	0.464328	3.388092	0.000000
1	1.549345	3.332084	0.000000
1	0.245505	4.462771	0.000000
6	-0.010698	2.900617	-1.419327
1	0.649377	3.434864	-2.111838
1	-0.995325	3.313252	-1.626406
6	2.506660	1.459573	1.419327
1	3.299368	1.155055	2.111838
1	2.371698	2.518603	1.626406
6	3.166338	1.291927	0.000000
1	3.660342	0.324270	0.000000
1	3.987626	2.018772	0.000000
6	2.506660	1.459573	-1.419327
1	3.299368	1.155055	-2.111838
1	2.371698	2.518603	-1.626406
6	2.517357	-1.441044	1.419327
1	2.649991	-2.279809	2.111838
1	3.367023	-0.794649	1.626406
6	2.702010	-2.096165	0.000000
1	2.110997	-3.007814	0.000000
1	3.742121	-2.443999	0.000000
6	2.517357	-1.441044	-1.419327
1	2.649991	-2.279809	-2.111838
1	3.367023	-0.794649	-1.626406
10	0.000000	0.000000	0.000000

Ar@[3_6](1,2,3,4,5,6)

6	-1.250458	-0.699762	1.981982
6	-0.028383	-1.420398	2.034641

6	-1.240706	0.717388	1.977243
6	-1.251140	-0.700243	-1.981434
6	-1.241389	0.717015	-1.976966
6	-0.029379	-1.420879	-2.034395
6	1.218319	0.705126	-2.079158
6	-0.010371	1.420768	-2.025057
6	1.209321	-0.721038	-2.084271
6	1.219078	0.705656	2.078794
6	1.210123	-0.720649	2.084206
6	-0.009437	1.421192	2.024770
6	-3.094574	-1.231323	0.000598
1	-3.567053	-0.255836	0.000594
1	-3.935191	-1.935973	0.000845
6	-2.499280	-1.427954	1.466039
1	-2.394348	-2.492212	1.661577
1	-3.345044	-1.131086	2.096100
6	-2.499857	-1.428236	-1.465018
1	-3.345787	-1.131425	-2.094880
1	-2.395014	-2.492548	-1.660425
6	-2.491974	1.441494	1.463179
1	-2.657586	2.319439	2.097001
1	-3.362496	0.818418	1.653707
6	-2.615411	2.059046	0.000234
1	-2.000943	2.951907	-0.000037
1	-3.643770	2.440669	0.000361
6	-2.492524	1.441126	-1.462587
1	-2.658487	2.318877	-2.096587
1	-3.363091	0.817952	-1.652608
6	-0.004172	2.859479	1.481251
1	-0.976340	3.308760	1.669694
1	0.680689	3.453890	2.094722
6	0.461633	3.256285	-0.000471
1	1.541754	3.171155	-0.000823
1	0.277791	4.337447	-0.000570
6	-0.005140	2.859134	-1.481776
1	0.678930	3.453759	-2.095901
1	-0.977685	3.307971	-1.669434
6	2.460483	1.421369	1.503959
1	3.332915	1.124962	2.094586
1	2.368607	2.487899	1.693953
6	3.017928	1.219939	-0.000565
1	3.492196	0.246090	-0.000551
1	3.856227	1.926781	-0.000777
6	2.459940	1.421027	-1.504898
1	3.332166	1.124566	-2.095787
1	2.367969	2.487512	-1.695089
6	2.454953	-1.429772	1.506520
1	2.637129	-2.335228	2.094053
1	3.330863	-0.815461	1.698779
6	2.562527	-2.010281	-0.000183
1	1.962153	-2.911860	0.000234
1	3.595945	-2.376858	-0.000253
6	2.454216	-1.430457	-1.506993
1	2.635698	-2.336213	-2.094283
1	3.330258	-0.816608	-1.700114
6	-0.016784	-2.858160	1.485695
1	-0.703371	-3.457661	2.093055
1	0.955322	-3.303924	1.680066
6	-0.475471	-3.255815	0.000294
1	-1.555949	-3.178605	0.000780
1	-0.284799	-4.335807	0.000477
6	-0.017961	-2.858626	-1.485455
1	-0.705457	-3.457766	-2.092170

1	0.953678	-3.304952	-1.680817
18	0.212081	-0.019041	-0.000030

Kr@[3_6](1,2,3,4,5,6)

6	1.322145	-0.535764	2.117016
6	1.127748	0.874744	2.113859
6	0.196948	-1.409223	2.122191
6	1.321806	-0.535742	-2.116928
6	0.196787	-1.409996	-2.121956
6	1.127322	0.874936	-2.114305
6	-1.317125	0.539540	-2.122955
6	-1.123020	-0.872375	-2.124637
6	-0.191650	1.412656	-2.116927
6	-1.316757	0.540148	2.122853
6	-0.191052	1.412720	2.116914
6	-1.122835	-0.871414	2.124681
6	2.817443	-1.617501	-0.000238
1	2.345315	-2.591595	-0.000295
1	3.891103	-1.840600	-0.000252
6	2.641143	-1.059670	1.509337
1	3.425017	-0.329920	1.695192
1	2.955826	-1.933048	2.089302
6	2.641094	-1.059369	-1.509670
1	2.955778	-1.932479	-2.090020
1	3.424669	-0.329300	-1.695457
6	0.402473	-2.813040	1.512098
1	-0.197138	-3.523337	2.089973
1	1.426452	-3.127198	1.697453
6	0.007144	-3.242201	0.000521
1	-1.072712	-3.317409	0.000447
1	0.348292	-4.284363	0.000655
6	0.402765	-2.813518	-1.511114
1	-0.196310	-3.524262	-2.089012
1	1.426982	-3.127200	-1.696036
6	-2.235962	-1.751989	1.513680
1	-1.997364	-2.795864	1.700723
1	-3.151528	-1.586067	2.090018
6	-2.804608	-1.626508	0.000169
1	-3.413006	-0.731088	-0.000044
1	-3.533651	-2.445654	0.000471
6	-2.235749	-1.753096	-1.513162
1	-3.151295	-1.588672	-2.089951
1	-1.996036	-2.796945	-1.698988
6	-2.636224	1.062703	1.512060
1	-2.951440	1.937198	2.089926
1	-3.419859	0.332940	1.698315
6	-2.811915	1.618464	-0.000211
1	-2.341707	2.593528	-0.000568
1	-3.885982	1.839786	-0.000208
6	-2.636674	1.061785	-1.512075
1	-2.952940	1.935523	-2.090507
1	-3.419801	0.331219	-1.697452
6	-0.398667	2.817182	1.509503
1	0.199681	3.526681	2.089828
1	-1.423024	3.129475	1.695443
6	-0.002826	3.248229	0.000003
1	1.077106	3.322432	-0.000132
1	-0.343266	4.290648	0.000150
6	-0.399213	2.817131	-1.509338
1	0.198861	3.526573	-2.089965

1	-1.423661	3.129385	-1.694815
6	2.241291	1.755902	1.508409
1	3.154234	1.590316	2.089255
1	2.001356	2.799525	1.694958
6	2.813529	1.629136	-0.000230
1	3.419329	0.731883	-0.000395
1	3.545286	2.445930	-0.000393
6	2.241020	1.756119	-1.508774
1	3.153785	1.590791	-2.090032
1	2.000570	2.799707	-1.694915
36	-0.014922	-0.009680	-0.000203

He@[3_5](1,2,3,4,5) – He@**3a**

6	1.676574	-0.066742	1.869588
6	1.082107	1.178524	1.687563
6	0.970021	-1.258657	1.720088
6	1.676574	-0.066742	-1.869588
6	0.970021	-1.258657	-1.720088
6	1.082107	1.178524	-1.687563
6	-1.071047	0.048950	-1.568683
6	-0.426261	-1.207452	-1.595214
6	-0.319691	1.242765	-1.577279
6	-1.071047	0.048950	1.568683
6	-0.319691	1.242765	1.577279
6	-0.426261	-1.207452	1.595214
6	1.790794	-2.492244	1.382378
1	1.206965	-3.408744	1.475458
1	2.595960	-2.581564	2.120631
6	2.517793	-2.443085	0.000000
1	3.214290	-3.289658	0.000000
1	3.143024	-1.543658	0.000000
6	1.790794	-2.492244	-1.382378
1	1.206965	-3.408744	-1.475458
1	2.595960	-2.581564	-2.120631
6	-1.210929	-2.488686	1.364723
1	-0.899169	-3.220860	2.118317
1	-2.265842	-2.315907	1.571614
6	-1.062225	-3.213087	0.000000
1	-1.800119	-4.023910	0.000000
1	-0.095796	-3.713679	0.000000
6	-1.210929	-2.488686	-1.364723
1	-2.265842	-2.315907	-1.571614
1	-0.899169	-3.220860	-2.118317
6	-2.577051	0.121253	1.361420
1	-2.921189	1.131501	1.573771
1	-3.065681	-0.497317	2.122683
6	-3.160596	-0.344509	0.000000
1	-4.217015	-0.052198	0.000000
1	-3.171506	-1.433127	0.000000
6	-2.577051	0.121253	-1.361420
1	-2.921189	1.131501	-1.573771
1	-3.065681	-0.497317	-2.122683
6	-0.985803	2.592878	1.359168
1	-0.260293	3.377008	1.571567
1	-1.759834	2.728893	2.123494
6	-1.676958	2.880086	0.000000
1	-1.930254	3.946575	0.000000
1	-2.636559	2.365103	0.000000
6	-0.985803	2.592878	-1.359168
1	-0.260293	3.377008	-1.571567

1	-1.759834	2.728893	-2.123494
6	2.005150	2.348509	1.370862
1	3.033509	1.986402	1.465430
1	1.900681	3.135258	2.128700
6	1.867247	3.071873	0.000000
1	2.655902	3.833618	0.000000
1	0.938906	3.638628	0.000000
6	2.005150	2.348509	-1.370862
1	3.033509	1.986402	-1.465430
1	1.900681	3.135258	-2.128700
1	2.761692	-0.118180	1.920932
1	2.761692	-0.118180	-1.920932
2	0.563796	-0.048755	0.000000

He@[3_4](1,2,3,5) – (He@**4a**)

6	1.078895	1.051594	1.854449
6	-0.139001	1.725869	1.778966
6	1.162617	-0.329934	1.694212
6	1.078895	1.051594	-1.854449
6	1.162617	-0.329934	-1.694212
6	-0.139001	1.725869	-1.778966
6	-1.257513	-0.439465	-1.650203
6	-0.016182	-1.100477	-1.588613
6	-1.291840	0.952890	-1.783650
6	-1.257513	-0.439465	1.650203
6	-1.291840	0.952890	1.783650
6	-0.016182	-1.100477	1.588613
6	2.542083	-0.882974	1.374553
1	2.579413	-1.967398	1.479919
1	3.243078	-0.494273	2.121769
6	3.129855	-0.441583	0.000000
6	2.542083	-0.882974	-1.374553
1	2.579413	-1.967398	-1.479919
1	3.243078	-0.494273	-2.121769
6	0.066703	-2.597385	1.336762
1	0.682031	-3.042509	2.127766
1	-0.917958	-3.044063	1.467382
6	0.652026	-3.096483	0.000000
6	0.066703	-2.597385	-1.336762
1	-0.917958	-3.044063	-1.467382
1	0.682031	-3.042509	-2.127766
6	-2.590151	-1.101639	1.332965
1	-3.297778	-0.843812	2.128811
1	-2.520747	-2.189526	1.342015
6	-3.240478	-0.661749	0.000000
6	-2.590151	-1.101639	-1.332965
1	-3.297778	-0.843812	-2.128811
1	-2.520747	-2.189526	-1.342015
6	-0.183512	3.185961	1.368176
1	0.332906	3.799757	2.115310
1	-1.223890	3.528248	1.352181
6	0.492312	3.518228	0.000000
6	-0.183512	3.185961	-1.368176
1	0.332906	3.799757	-2.115310
1	-1.223890	3.528248	-1.352181
1	2.001499	1.628097	1.847807
1	2.001499	1.628097	-1.847807
1	-2.254251	1.454490	1.718915
1	-2.254251	1.454490	-1.718915
1	0.638070	4.604077	0.000000

1	1.498685	3.086251	0.000000
1	3.155543	0.653540	0.000000
1	4.177895	-0.762877	0.000000
1	1.724848	-2.921450	0.000000
1	0.542532	-4.187327	0.000000
1	-3.345301	0.427608	0.000000
1	-4.261534	-1.060034	0.000000
2	-0.023407	0.509207	0.000000

He@[3_4](1,2,4,5) – (He@**5a**)

6	0.000078	1.353252	1.826828
6	-1.227803	0.701069	1.695857
6	1.227808	0.700759	1.696008
6	-0.000068	1.354949	-1.826073
6	1.227883	0.702707	-1.695822
6	-1.227743	0.702345	-1.695423
6	0.000314	-1.353205	-1.827124
6	1.228047	-0.700696	-1.696149
6	-1.227577	-0.701069	-1.696151
6	-0.000270	-1.354896	1.826140
6	-1.227974	-0.702321	1.695434
6	1.227649	-0.702653	1.695756
6	2.433425	1.552690	1.341020
1	3.351927	0.962802	1.341118
1	2.565602	2.306846	2.125261
6	2.331495	2.324368	0.001180
6	2.433087	1.554875	-1.339952
1	3.352075	0.965740	-1.341737
1	2.564179	2.310469	-2.122996
6	2.432883	-1.554892	1.340267
1	3.351953	-0.965888	1.342327
1	2.563636	-2.310589	2.123262
6	2.331367	-2.324287	-0.000966
6	2.433533	-1.552701	-1.340855
1	2.565690	-2.306975	-2.124988
1	3.352117	-0.962936	-1.340974
6	-2.433432	-1.554122	1.339677
1	-3.352144	-0.964532	1.340715
1	-2.565160	-2.309232	2.123072
6	-2.331462	-2.324315	-0.001043
6	-2.432968	-1.553382	-1.341349
1	-3.351792	-0.963985	-1.342435
1	-2.564238	-2.308045	-2.125256
6	-2.433259	1.553287	1.341130
1	-2.564735	2.307780	2.125165
1	-3.351988	0.963727	1.341964
6	-2.331690	2.324373	0.000927
6	-2.433314	1.554090	-1.339751
1	-2.565114	2.309072	-2.123257
1	-3.351936	0.964381	-1.340774
1	0.000190	2.441300	1.825646
1	-0.000210	2.442994	-1.822536
1	-0.000408	-2.442950	1.824017
1	0.000483	-2.441257	-1.825650
1	-3.145287	-3.058370	-0.001377
1	-1.403725	-2.905848	-0.001017
1	-3.145652	3.058279	0.001145
1	-1.404079	2.906097	0.001121
1	1.403777	2.905920	0.001763
1	3.145329	3.058416	0.001594
1	1.403553	-2.905698	-0.001587

1	3.145093	-3.058453	-0.001345
2	0.000404	-0.000209	0.000186

Ar@[3_4](1,2,4,5) – (Ar@**5a**)

6	-2.349032	0.000000	1.360787
6	-2.118572	1.223564	0.704751
6	-2.118572	-1.223564	0.704751
6	2.349032	-0.000000	1.360787
6	2.118572	-1.223564	0.704751
6	2.118572	1.223564	0.704751
6	2.348389	-0.000000	-1.361060
6	2.118387	-1.223578	-0.704969
6	2.118387	1.223578	-0.704969
6	-2.348389	0.000000	-1.361060
6	-2.118387	1.223578	-0.704969
6	-2.118387	-1.223578	-0.704969
6	-1.426913	-2.320676	1.528038
1	-1.382086	-3.260373	0.974667
1	-2.067229	-2.524403	2.392790
6	0.000000	-2.031768	2.169860
6	1.426913	-2.320676	1.528038
1	1.382086	-3.260373	0.974667
1	2.067229	-2.524403	2.392790
6	-1.426581	-2.320724	-1.528216
1	-1.381208	-3.260140	-0.974444
1	-2.067229	-2.524987	-2.392600
6	0.000000	-2.031404	-2.170217
6	1.426581	-2.320724	-1.528216
1	2.067229	-2.524987	-2.392600
1	1.381208	-3.260140	-0.974444
6	-1.426581	2.320724	-1.528216
1	-1.381208	3.260140	-0.974444
1	-2.067229	2.524987	-2.392600
6	0.000000	2.031404	-2.170217
6	1.426581	2.320724	-1.528216
1	1.381208	3.260140	-0.974444
1	2.067229	2.524987	-2.392600
6	-1.426913	2.320676	1.528038
1	-2.067229	2.524403	2.392790
1	-1.382086	3.260373	0.974667
6	0.000000	2.031768	2.169860
6	1.426913	2.320676	1.528038
1	2.067229	2.524403	2.392790
1	1.382086	3.260373	0.974667
1	-2.298098	0.000000	2.448466
1	2.298098	-0.000000	2.448466
1	-2.297222	0.000000	-2.448740
1	2.297222	-0.000000	-2.448740
1	0.000000	2.672515	-3.057286
1	0.000000	1.011379	-2.561386
1	0.000000	2.673297	3.056499
1	0.000000	1.011855	2.561497
1	-0.000000	-1.011855	2.561497
1	-0.000000	-2.673297	3.056499
1	-0.000000	-1.011379	-2.561386
1	-0.000000	-2.672515	-3.057286
18	0.000000	0.000000	0.000960

Kr@[3_4](1,2,4,5) – (Kr@**5a**)

6	-2.433883	0.000000	1.363888
6	-2.184365	1.223099	0.706883
6	-2.184365	-1.223099	0.706883
6	2.433883	-0.000000	1.363888
6	2.184365	-1.223099	0.706883
6	2.184365	1.223099	0.706883
6	2.433472	-0.000000	-1.364239
6	2.184001	-1.223011	-0.707174
6	2.184001	1.223011	-0.707174
6	-2.433472	0.000000	-1.364239
6	-2.184001	1.223011	-0.707174
6	-2.184001	-1.223011	-0.707174
6	-1.450744	-2.307579	1.525228
1	-1.416161	-3.250648	0.976987
1	-2.068278	-2.508228	2.406933
6	0.000000	-2.029352	2.152636
6	1.450744	-2.307579	1.525228
1	1.416161	-3.250648	0.976987
1	2.068278	-2.508228	2.406933
6	-1.450921	-2.307612	-1.525475
1	-1.416838	-3.250955	-0.977696
1	-2.068291	-2.507585	-2.407458
6	-0.000000	-2.029515	-2.152664
6	1.450921	-2.307612	-1.525475
1	2.068291	-2.507585	-2.407458
1	1.416838	-3.250955	-0.977696
6	-1.450921	2.307612	-1.525475
1	-1.416838	3.250955	-0.977696
1	-2.068291	2.507585	-2.407458
6	0.000000	2.029515	-2.152664
6	1.450921	2.307612	-1.525475
1	1.416838	3.250955	-0.977696
1	2.068291	2.507585	-2.407458
6	-1.450744	2.307579	1.525228
1	-2.068278	2.508228	2.406933
1	-1.416161	3.250648	0.976987
6	0.000000	2.029352	2.152636
6	1.450744	2.307579	1.525228
1	2.068278	2.508228	2.406933
1	1.416161	3.250648	0.976987
1	-2.374175	0.000000	2.451226
1	2.374175	-0.000000	2.451226
1	-2.373437	0.000000	-2.451556
1	2.373437	-0.000000	-2.451556
1	-0.000000	2.700264	-3.017655
1	-0.000000	1.023601	-2.579625
1	0.000000	2.700414	3.017468
1	0.000000	1.023651	2.579969
1	-0.000000	-1.023651	2.579969
1	-0.000000	-2.700414	3.017468
1	-0.000000	-1.023601	-2.579625
1	-0.000000	-2.700264	-3.017655
36	0.000000	0.000000	0.000631

He@[3_3](1,2,3) – (He@**6a**)

6	0.300913	-1.281515	1.821073
6	1.301080	-0.308445	1.750448
6	-1.047694	-0.937850	1.730467
6	0.300913	-1.281515	-1.821073
6	-1.047694	-0.937850	-1.730467
6	1.301080	-0.308445	-1.750448
6	-0.417192	1.413182	-1.703173
6	-1.390235	0.415760	-1.724277
6	0.925461	1.033482	-1.765503
6	-0.417192	1.413182	1.703173
6	0.925461	1.033482	1.765503
6	-1.390235	0.415760	1.724277
6	-2.075404	-1.986804	1.349875
1	-3.070602	-1.529758	1.331872
1	-2.107777	-2.764620	2.121343
6	-1.823161	-2.714903	0.000000
1	-2.478929	-3.592584	0.000000
1	-0.801672	-3.109632	0.000000
6	-2.075404	-1.986804	-1.349875
1	-3.070602	-1.529758	-1.331872
1	-2.107777	-2.764620	-2.121343
6	-0.789812	2.837938	1.341354
1	-0.434468	3.517580	2.124425
1	-1.880928	2.931998	1.324973
6	-0.214707	3.363202	0.000000
1	0.870901	3.221359	0.000000
1	-0.370198	4.447682	0.000000
6	-0.789812	2.837938	-1.341354
1	-0.434468	3.517580	-2.124425
1	-1.880928	2.931998	-1.324973
6	2.713161	-0.702303	1.356487
1	3.136786	-1.366166	2.118886
1	3.347198	0.190559	1.338882
6	2.848833	-1.454384	0.000000
1	2.155593	-2.301944	0.000000
1	3.851886	-1.894993	0.000000
6	2.713161	-0.702303	-1.356487
1	3.136786	-1.366166	-2.118886
1	3.347198	0.190559	-1.338882
1	0.581939	-2.330009	1.757899
1	0.581939	-2.330009	-1.757899
1	1.695451	1.792682	1.654781
1	1.695451	1.792682	-1.654781
1	-2.430973	0.692980	1.577674
1	-2.430973	0.692980	-1.577674
2	-0.004973	-0.002613	0.000000

Ne@[3_3](1,2,3) – (Ne@**6a**)

6	-0.414569	-1.332222	-1.967635
6	-1.374737	-0.310731	-1.920347
6	0.948695	-1.045986	-1.929407
6	-0.417969	-1.327218	1.970032
6	0.945339	-1.040889	1.933482
6	-1.378212	-0.306005	1.918659
6	0.419812	1.338042	1.951093
6	1.348841	0.300525	2.020297
6	-0.945473	1.016229	2.002553

6	0.423634	1.332976	-1.953478
6	-0.941611	1.011232	-2.006301
6	1.352577	0.295160	-2.018708
6	1.923988	-2.082008	-1.381840
1	2.756300	-2.203108	-2.084109
1	1.425465	-3.055368	-1.327504
6	2.590285	-1.760408	0.004381
1	2.901878	-0.711476	0.003217
1	3.520717	-2.337196	0.005929
6	1.921668	-2.078295	1.390318
1	2.752771	-2.197553	2.094335
1	1.423236	-3.051798	1.337711
6	0.843808	2.690519	-1.396072
1	0.535780	3.480042	-2.090587
1	1.936503	2.737625	-1.344963
6	0.236983	3.106001	-0.003706
1	-0.827963	2.855897	-0.004570
1	0.274525	4.199958	-0.005180
6	0.840771	2.694318	1.391109
1	0.531117	3.485580	2.082917
1	1.933561	2.741527	1.342219
6	-2.767722	-0.621037	-1.384784
1	-3.290619	-1.274185	-2.092591
1	-3.350232	0.304923	-1.336830
6	-2.846890	-1.356521	-0.000789
1	-2.117175	-2.171578	0.000833
1	-3.825638	-1.847212	-0.001123
6	-2.770256	-0.617703	1.381543
1	-3.294317	-1.269110	2.090085
1	-3.352682	0.308134	1.330315
1	-0.731854	-2.357216	-1.794039
1	-0.734800	-2.352683	1.798390
1	-1.671078	1.803727	-1.859493
1	-1.674843	1.808255	1.852824
1	2.403761	0.533642	-1.876858
1	2.400229	0.538866	1.879717
10	0.044158	0.059444	-0.000305

Ar@[3_3](1,2,3) – (Ar@**6a**)

6	-2.276974	1.273494	-0.561755
6	-2.189385	0.151784	-1.402919
6	-2.188308	1.140182	0.830203
6	2.277182	1.273409	-0.560706
6	2.187849	1.140175	0.831208
6	2.190020	0.151637	-1.401901
6	2.191116	-1.288302	0.570657
6	2.278319	-0.149015	1.381974
6	2.281677	-1.120536	-0.821295
6	-2.191501	-1.288293	0.569664
6	-2.281050	-1.120440	-0.822275
6	-2.279128	-0.148982	1.380936
6	-1.437656	2.227366	1.630094
1	-2.043968	2.517357	2.493920
1	-1.348027	3.123646	1.008138
6	-0.000544	1.872256	2.252581
1	-0.000592	0.818733	2.544494
1	-0.000745	2.422752	3.196839
6	1.436817	2.227400	1.630686
1	2.042740	2.517519	2.494736
1	1.347384	3.123592	1.008573

6	-1.439614	-2.524326	1.111899
1	-2.044562	-3.418035	0.929536
1	-1.352054	-2.434208	2.199267
6	-0.000212	-2.887236	0.496482
1	-0.000004	-2.619849	-0.563578
1	-0.000225	-3.980238	0.508028
6	1.438930	-2.524330	1.112563
1	2.043926	-3.418099	0.930681
1	1.350765	-2.434073	2.199873
6	-1.436495	0.299472	-2.743716
1	-2.041542	0.901490	-3.428787
1	-1.345837	-0.687930	-3.207340
6	0.000739	1.016479	-2.743859
1	0.000577	1.790893	-1.972457
1	0.000990	1.564727	-3.689375
6	1.438055	0.299485	-2.743071
1	2.043497	0.901650	-3.427664
1	1.347577	-0.687820	-3.206940
1	-2.056273	2.248451	-0.990328
1	2.056904	2.248380	-0.989457
1	-2.062841	-1.979567	-1.452593
1	2.063830	-1.979669	-1.451718
1	-2.059869	-0.265480	2.439870
1	2.058490	-0.265521	2.440792
18	0.000046	-0.007709	0.000044

Kr@[3_3](1,2,3) – (Kr@**6a**)

6	2.358595	1.056693	0.906343
6	2.262801	-0.259748	1.389787
6	2.262834	1.332632	-0.468942
6	-2.358585	1.056694	0.906346
6	-2.262826	1.332638	-0.468921
6	-2.262800	-0.259742	1.389765
6	-2.263843	-1.073236	-0.918975
6	-2.360826	0.255909	-1.367224
6	-2.359264	-1.312633	0.462907
6	2.263842	-1.073242	-0.918969
6	2.359264	-1.312637	0.462936
6	2.360822	0.255908	-1.367248
6	1.466301	2.592245	-0.911864
1	2.047905	3.125786	-1.669886
1	1.388739	3.271135	-0.056800
6	-0.000001	2.450615	-1.595160
1	-0.000006	1.563254	-2.233942
1	-0.000003	3.293411	-2.291679
6	-1.466284	2.592254	-0.911829
1	-2.047900	3.125819	-1.669826
1	-1.388702	3.271122	-0.056750
6	1.466640	-2.086304	-1.788315
1	2.047649	-3.009894	-1.871361
1	1.388951	-1.685350	-2.803787
6	-0.000004	-2.606452	-1.323018
1	-0.000007	-2.712407	-0.234818
1	-0.000006	-3.632161	-1.701745
6	-1.466638	-2.086292	-1.788328
1	-2.047653	-3.009877	-1.871389
1	-1.388938	-1.685328	-2.803795
6	1.466172	-0.506008	2.701912
1	2.047531	-0.116361	3.543157
1	1.388375	-1.585933	2.862302

6	-0.000003	0.157578	2.919207
1	-0.000001	1.152652	2.466116
1	-0.000007	0.343598	3.996602
6	-1.466175	-0.506006	2.701892
1	-2.047541	-0.116378	3.543141
1	-1.388370	-1.585932	2.862263
1	2.121124	1.863589	1.596359
1	-2.121109	1.863589	1.596362
1	2.121583	-2.313726	0.816432
1	-2.121597	-2.313730	0.816388
1	2.124345	0.450182	-2.411254
1	-2.124355	0.450179	-2.411232
36	-0.000004	-0.000346	-0.002352

He@[3_3](1,2,3) – (He@**6b**)

6	0.300913	-1.281515	1.821073
6	1.301080	-0.308445	1.750448
6	-1.047694	-0.937850	1.730467
6	0.300913	-1.281515	-1.821073
6	-1.047694	-0.937850	-1.730467
6	1.301080	-0.308445	-1.750448
6	-0.417192	1.413182	-1.703173
6	-1.390235	0.415760	-1.724277
6	0.925461	1.033482	-1.765503
6	-0.417192	1.413182	1.703173
6	0.925461	1.033482	1.765503
6	-1.390235	0.415760	1.724277
6	-2.075404	-1.986804	1.349875
1	-3.070602	-1.529758	1.331872
1	-2.107777	-2.764620	2.121343
6	-1.823161	-2.714903	0.000000
1	-2.478929	-3.592584	0.000000
1	-0.801672	-3.109632	0.000000
6	-2.075404	-1.986804	-1.349875
1	-3.070602	-1.529758	-1.331872
1	-2.107777	-2.764620	-2.121343
6	-0.789812	2.837938	1.341354
1	-0.434468	3.517580	2.124425
1	-1.880928	2.931998	1.324973
6	-0.214707	3.363202	0.000000
1	0.870901	3.221359	0.000000
1	-0.370198	4.447682	0.000000
6	-0.789812	2.837938	-1.341354
1	-0.434468	3.517580	-2.124425
1	-1.880928	2.931998	-1.324973
6	2.713161	-0.702303	1.356487
1	3.136786	-1.366166	2.118886
1	3.347198	0.190559	1.338882
6	2.848833	-1.454384	0.000000
1	2.155593	-2.301944	0.000000
1	3.851886	-1.894993	0.000000
6	2.713161	-0.702303	-1.356487
1	3.136786	-1.366166	-2.118886
1	3.347198	0.190559	-1.338882
1	0.581939	-2.330009	1.757899
1	0.581939	-2.330009	-1.757899
1	1.695451	1.792682	1.654781
1	1.695451	1.792682	-1.654781
1	-2.430973	0.692980	1.577674
1	-2.430973	0.692980	-1.577674
2	-0.004973	-0.002613	0.000000

Values of ρ and $\nabla^2\rho$ at BCPs

Table S1. Values of ρ and $\nabla^2\rho$ (in a.u.) at BCPs of Ng···C_r , Ng···π, and Ng···C_c interactions.

Complex	BCP					
	Ng···C_r		Ng···π		Ng···C_c	
	ρ	$\nabla^2\rho$			ρ	$\nabla^2\rho$
He@[3_6]SP	0.0298	0.1566	n/a	n/a	n/a	n/a
Ne@[3_6]SP	0.0340	0.1813	n/a	n/a	n/a	n/a
Ar@[3_6]SP	0.0437	0.1857	n/a	n/a	0.0116	0.0472
Kr@[3_6]SP	0.0415	0.1534	n/a	n/a	0.0108	0.0399
He@**3a**	0.0278	0.1453	n/a	n/a	n/a	n/a
He@**4a**	0.0264	0.1374	n/a	n/a	n/a	n/a
He@**5a**	n/a	n/a	0.0251	0.1299	n/a	n/a
Ar@**5a**	n/a	n/a	0.0347	0.1436	0.0130	0.0568
Kr@**5a**	n/a	n/a	0.0385	0.1347	0.0169	0.0650
He@**6a**	0.0234	0.1252	n/a	n/a	n/a	n/a
Ne@**6a**	0.0271	0.1375	n/a	n/a	n/a	n/a
Ar@**6a**	0.0292	0.1257	n/a	n/a	0.0144	0.0622
Kr@**6a**	0.0318	0.1193	n/a	n/a	0.0181	0.0686
He@**6b**	0.0238	0.1264	n/a	n/a	n/a	n/a
